# Tracking the Multistep Formation of Ln(III) Complexes with *in situ* Schiff Base Exchange Reaction and its Highly Selective Sensing of Dichloromethane

**DOI:** 10.1038/s41598-019-48696-y

**Published:** 2019-08-22

**Authors:** Kai-Qiang Mo, Xiong-Feng Ma, Hai-Ling Wang, Zhong-Hong Zhu, Yan-Cheng Liu, Hua-Hong Zou, Fu-Pei Liang

**Affiliations:** State Key Laboratory for Chemistry and Molecular Engineering of Medicinal Resources, School of Chemistry & Pharmacy of Guangxi Normal University, Guilin, 541004 P.R. China

**Keywords:** Environmental sciences, Chemistry

## Abstract

Four complexes, namely, [Ln_2_(**L2**)_2_(NO_3_)_4_]. 2CH_3_OH (Ln = Tb (**1**), Dy (**2**), Ho (**3**), Er (**4**), and **L2** = (*E*)-2-methoxy-6-(((pyridin-2-ylmethyl)imino)methyl)phenol), were obtained by reacting (*E*)-2-((3-methoxy-2-oxidobenzylidene)amino)ethanesulfonate (**L1**), Ln(NO_3_)_3_·6H_2_O, and 2-aminomethylpyridine at room temperature under solvothermal conditions in methanol for 12 h. The new Schiff base **L2** was generated *in situ* based on the organic ligand **L1** and 2-aminomethylpyridine through Schiff base exchange reaction by using lanthanide salts as inductor. A combination of crystallography and mass spectrometry was performed to track the exchange reaction, and the underlying mechanism accompanied by the complex assembly process was clearly presented. The multistep formation mechanism of the above dinuclear complex was also proposed, i.e., [**L1**] → Dy[**L1**]/[**L2**] → Dy[**L2**] → Dy[**L2**]_2_ → Dy_2_[**L2**]_2_. Luminescence test of **1** showed that it had extremely high selectivity to dichloromethane (CH_2_Cl_2_). Therefore, we established a quick, simple, and efficient method of detecting CH_2_Cl_2_ that enabled strong-luminescence observation with the naked eye. Tests for small amounts of CH_2_Cl_2_ in water further indicated the potential of **1** as a test strip for CH_2_Cl_2_ fluorescence detection in water samples. Alternating-current magnetic susceptibility studies indicated the field-induced single-molecule magnet behavior of **2**.

## Introduction

Life originates from solution, and the complex, variable chemical reactions occurring in solution often obey laws that are difficult to predict^1–6^. Science has raised a major scientific problem, “How far can we push chemical self-assembly?”^5,6^. Accordingly, an important goal in chemistry is to understand and determine the precise mechanism of chemical reactions to achieve precise control of chemical reactions^7–16^. Complexes are usually synthesized under solvothermal conditions at certain temperatures and pressures, thereby conferring difficulty in the real-time monitoring of the reaction process and assembly mechanism^7,8,12–16^. Thus, reactions occurring under normal temperature and pressure conditions are beneficial to monitoring these reactions’ processes and determining their mechanism. Schiff bases are the most widely used organic ligands in coordination chemistry due to their strong coordination ability, abundant coordination sites, and high coordination selectivity^17,18^. Schiff bases are generally stable during the formation of complexes^17,18^. Therefore, the identification and real-time monitoring of the *in situ* exchange reaction of Schiff base ligands in are difficult during complex self-assembly^19^. because Schiff base exchange reactions increase the number of species in a solution.

Research on process mechanisms is equally important as that on compound functions^20,21^. The “Holy Grail in Chemistry” emphasizes the importance of advancing research on ultrasensitive molecules for detection, which is a huge challenge^22^. Over the past few years, interest in finding highly selective and sensitive approaches to detecting an organic molecule with multitudes of potential health and environmental impacts has increased^23–27^. Among such organic molecules is dichloromethane (CH_2_Cl_2_), which is one of the most commonly used organic solvents in chemical production, film production, and pharmaceutical fields; however, its large emissions lead to water and air pollution^28^. The detection of CH_2_Cl_2_ still relies on gas chromatography, a quasi-quantitative detection technology that requires considerable time, labor, and cost. Therefore, for certain specific environments and the rational allocation of resources, developing a real-time, rapid detection method for CH_2_Cl_2_ is urgent. The design, synthesis, and development of selective and sensitive organic molecule sensors are research hotspots^23–27^. although the rapid detection of many organic molecules such as tetrahydrofuran (THF)^27^. and acetonitrile^26^. has been achieved. However, methods with high selectivity and fast response to CH_2_Cl_2_ remain necessary to establish.

Herein, we achieved an *in situ* Schiff base exchange reaction between (*E*)-2-((3-methoxy-2-oxidobenzylidene)amino)ethanesulfonate (**L1**) and 2-aminomethylpyridine induced by Ln(III) ions at room temperature to obtain [Ln_2_(**L2**)_2_(NO_3_)_4_] · 2CH_3_OH (Ln = Tb (**1**), Dy (**2**), Ho (**3**), and Er (**4**); **L2** = (*E*)-2-methoxy-6-(((pyridin-2-ylmethyl)imino)methyl)phenol). We used a combination of crystallography and electrospray ionization mass spectrometry (ESI-MS) to track the multistep assembly process of the above-described dinuclear complexes and proposed its assembly mechanism. The Schiff base exchange mechanism accompanying the complex assembly process was tracked and proposed (Table S1). This method can serve as an invaluable reference for mechanistic research (Tables S1–S2). Complexes **1** and **2**, dissolved in *N*,*N*-dimethylformamide (DMF), showed π–π* level transitions between ligands, as well as energy-level transitions from metal-ion-to-ligand charge transfer (MLCT). When **1** was dissolved in a different solvent to test its luminescence, it showed strong fluorescence in CH_2_Cl_2_ and weak fluorescence in other solvents. When **1** was dissolved and dispersed in different organic solvents under a UV lamp, only CH_2_Cl_2_ solution showed strong luminescence. Thus, the method was a rapid, easy, and effective way to detect CH_2_Cl_2_.

## Results and Discussion

### Single-crystal X-ray diffraction studies

**Figure 1:**
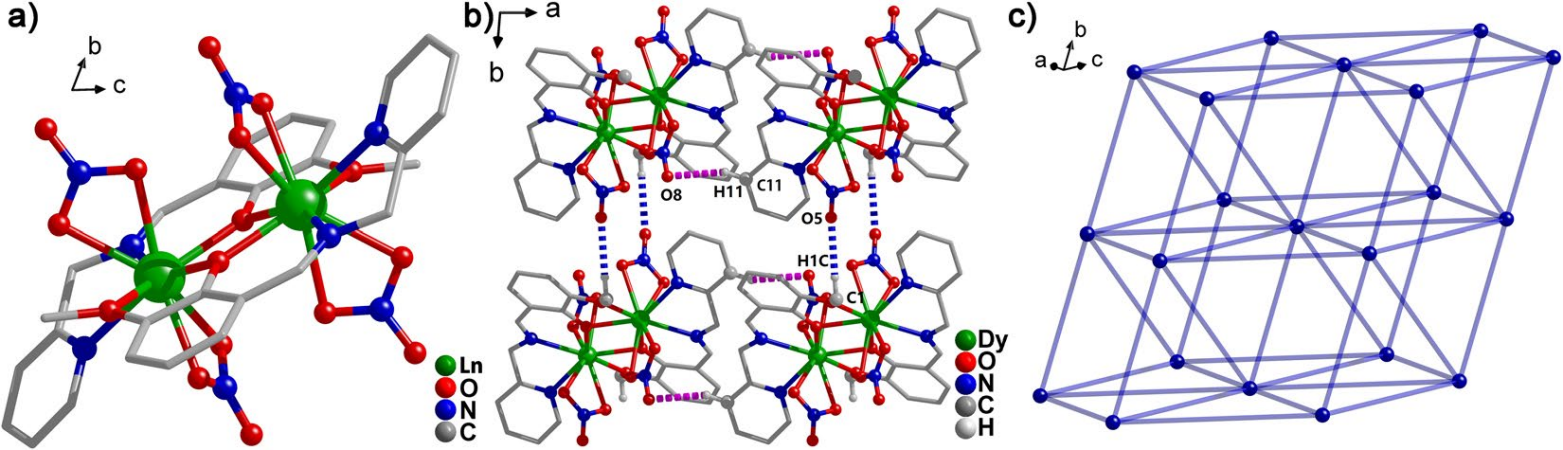
(**a**) Crystal structure for **Ln2** with most H atoms omitted for clarity. (**b**) Structure of **Ln2** showing the supramolecular interactions: same type ${\rm{C}}1-{\rm{H}}1{\rm{C}}\cdots {\rm{O}}5$ (blue dotted line) and ${\rm{C}}11-{\rm{H}}11\cdots {\rm{O}}8$ (pink dotted line) hydrogen bonds. (**c**) Connecting modes of clusters through hydrogen bonds in **Ln2** (Ln = Dy, Tb, Ho, Er).

X-ray diffraction results suggested that **1**, **2**, **3**, and **4** crystallized in the triclinic crystal system with the *P*-1 space group (Table S3), in which the nine-coordinated (N_2_O_7_) Ln(III) cores were surrounded by two N atoms, two *µ*_2_-O^−^ ions, one O atom from two **L2**, and two bidentate chelating nitrate anions (Fig. 1a). The intracluster Ln(III) ions were bridged by two phenoxo oxygen atoms from **L2** ligands. The bond length distances of Ln-O varied from 2.308(2) to 2.501(3) Å for **1**, from 2.298(2) to 2.488(3) Å for **2**, from 2.292(2) to 2.478(3) Å for **3**, and from 2.288(3) to 2.494(4) Å for **4**. The average Ln-O bond lengths were 2.436, 2.423, 2.411, and 2.407 Å for **1**, **2**, **3**, and **4**, respectively. The Ln-N bond distances ranged within 2.439(3)–2.522(3) Å for **1**, 2.426(3)–2.516(3) Å for **2**, 2.411(3)–2.501(3) Å for **3**, and 2.401(4)–2.494(4) Å for **4** (Table S4). The intracluster Ln∙∙∙Ln distances were 3.741, 3.730, 3.719, and 3.713 Å for **1**, **2**, **3**, and **4**, respectively. By studying the weak interactions among the **Ln2** molecules, only two of the same type of weak interaction, i.e., C-H∙∙∙O hydrogen bonding, were found (Fig. 1b); however, for all compounds, a slight difference in distance was observed (Table S5).The connecting modes of the clusters through hydrogen bonds and the above-mentioned supramolecular weak-action distances were all within a logical range. Thus, they can be considered as a formation of 10-connected ***fcu*** net (Fig. 1c) with distances of 9.976–12.590 Å for **1**, 9.972–12.572 Å for **2**, 9.947–12.531 Å for **3**, and 9.944–12.527 Å for **4** between the centers of the **Ln2** dimer. By using *SHAPE*, the calculated results suggested that the geometry of the nine-coordinated Ln(III) was a muffin for **Ln2** (Tables S6–S9). The TGA curves of the four compounds are shown in Fig. S1. The phase purities of **1**, **2**, **3**, and **4** were determined from their powder X-ray diffraction patterns (Fig. S2).

**Figure 2:**
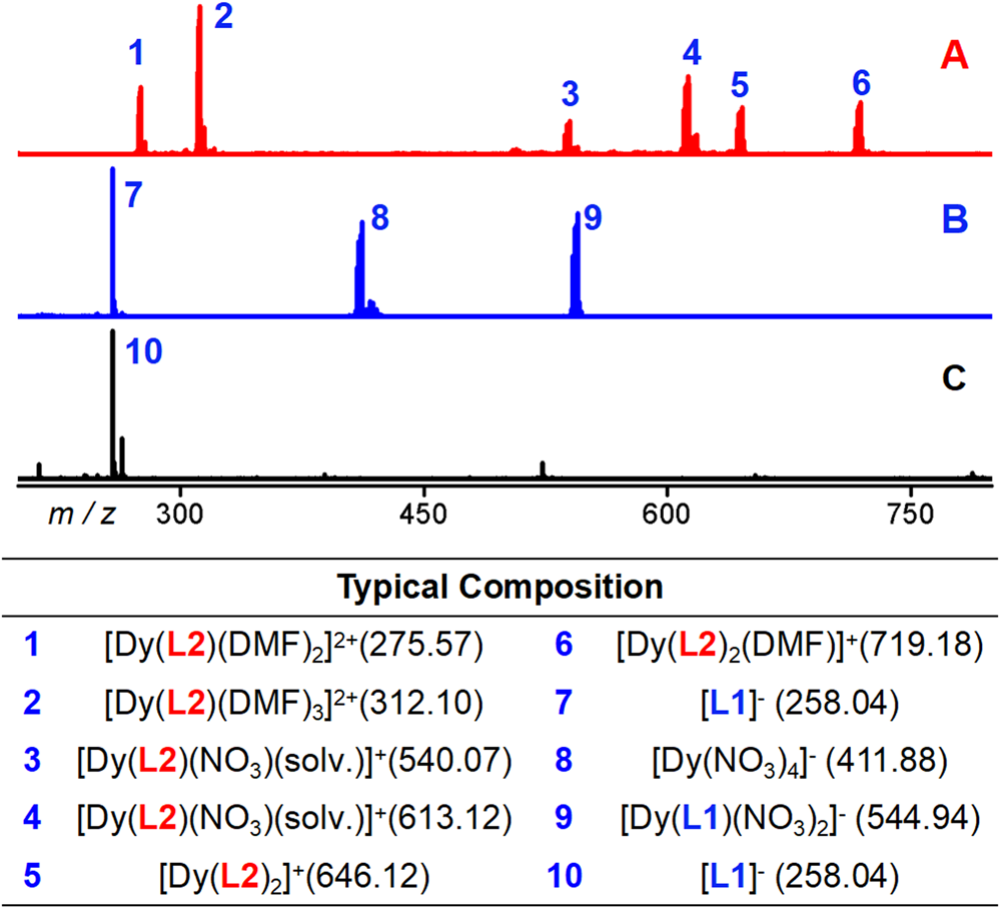
Top: (**A**) ESI-MS spectrum of crystal **2** in positive mode. (**B**) **L1** + Dy(NO_3_)_3_·6H_2_O was reacted in methanol at room temperature for 24 h (no added 2-aminomethylpyridine; negative mode). (**C**) **L1** + 2-aminomethylpyridine was reacted in methanol at room temperature for 24 h (no added Dy(NO_3_)_3_·6H_2_O; negative mode). Bottom: major species assigned in the ESI-MS.

**Figure 3:**
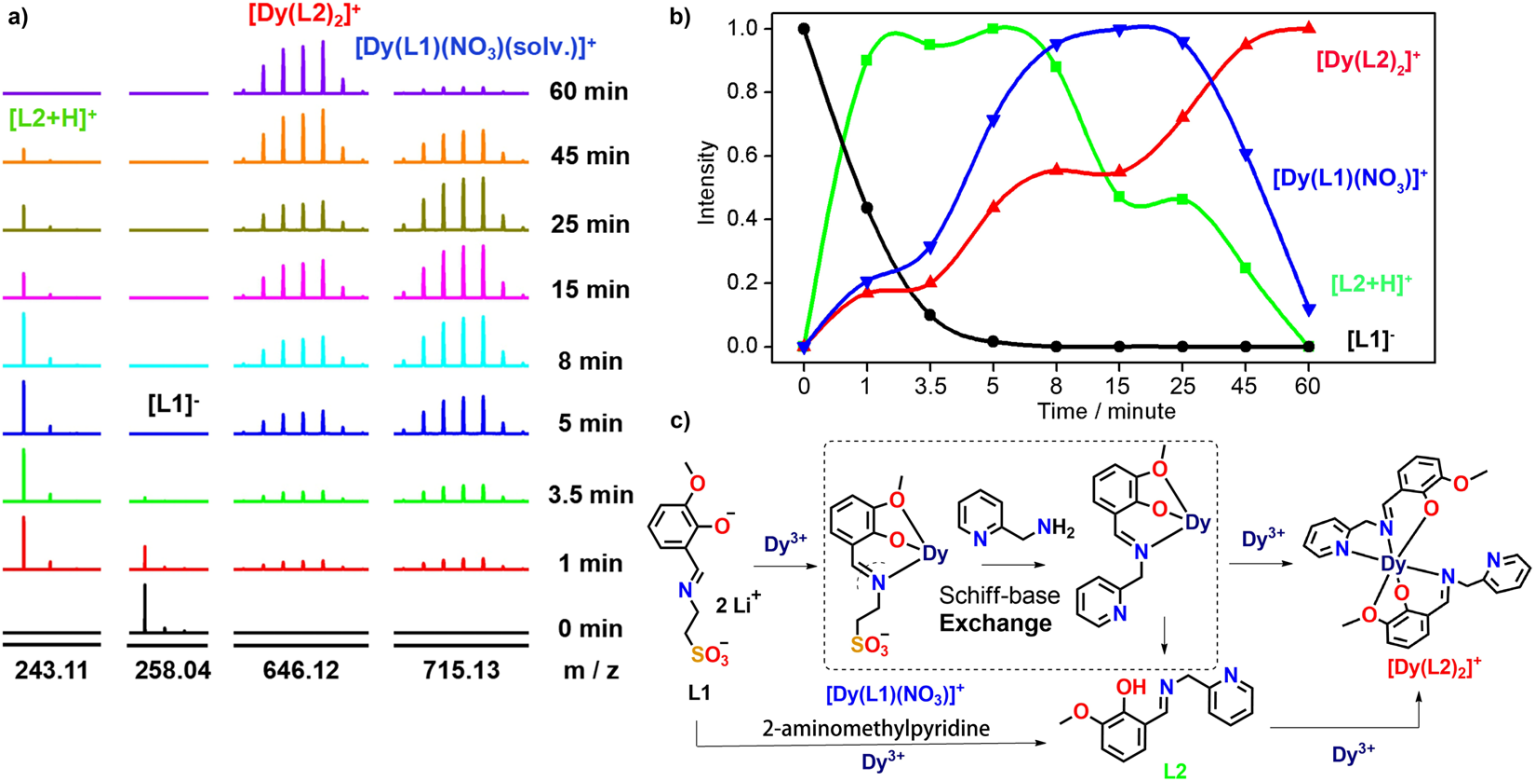
(**a**) Time-dependent ESI-MS tracks the formation of **2** and the Schiff base exchange process. (**b**) The tendency of fragments appearing during the reaction to change over time. (**c**) A **2** multistep assembly mechanism comprising a Schiff base exchange reaction (solv. = (DMF)_2_(CH_3_OH)(H_2_O)_3_). NO_3_^−^ ions are omitted for clarity.

To study the stability of the dinuclear complexes in solution, we performed ESI-MS tests. For the mass spectra of **1**, **2**, **3**, and **4**, their peak location and frame peaks were similar, so only the details of **2** were provided in the ensuing discussion. According to the correlation between the solid and liquid structures, at *m/z* = 993.00 (the main frame peak of the structure), the molecular formula [Dy_2_(**L2**)_2_(NO_3_)_3_]^+^ (*calc*. 993.01) was obtained by analysis and fitting. However, its peak strength was <0.1, indicating that **2** was unstable based on the mass spectrum in solution. A similar peak appeared at *m/z* = 1173.12. [Dy_2_(**L2**)_3_(NO_3_)_2_]^+^ (*calc*. 1173.12) can be obtained by fitting, indicating that with a [**L2**]^−^ ligand instead of a NO_3_^−^ anion, its strength was lower. The highest intensity peak appeared at *m/z* = 312.09, and the bivalent peak of [Dy(**L2**)(DMF)_3_]^2+^ (*calc*.312.09) was obtained by fitting. Similar fragment peaks appeared at *m/z* = 275.56 and 348.62, and the molecular fragments showed divalence, which differed from the number of DMF. This finding indicated that **2** was broken into the half-structure of Dy**L2** under mass-spectrometry condition, and DMF replaced the position of the NO_3_^−^anion and showed a +2 valence. Moreover, at *m/z* = 540.06 and 613.12, [Dy(**L2**)(NO_3_)(DMF)]^+^ (*calc*.540.06) and [Dy(**L2**)(NO_3_)(DMF)_2_]^+^ (*calc*.613.12) were obtained by fitting, and their intensities were 0.221 and 0.526, respectively. At *m/z* = 646.12 and 719.17, these miframe changed, the molecular formulas [Dy(**L2**)_2_]^+^ (*calc*.646.12) and [Dy(**L2**)_2_(DMF)]^+^ (*calc*.719.17) were obtained by fitting, and a [**L2**]^−^ ligand was found to replace a NO_3_^−^ anion (strength >0.3). Overall, under the ESI-MS condition, the above dinuclear complexes were more prone to homolysis to produce Dy**L2** semistructural fragments, and only a small amount of dinuclear structure lost one Dy(III) ion to produce Dy(**L2**)_2_ fragments (Fig. 2). Although the binuclear framework of **1**, **2**, **3**, and **4** was unstable under ESI-MS conditions, **L2** in the above complexes was always stable (Figs S3–S13, Tables S10 and S11). The ESI-MS measurements for control experiments were performed in negative mode (Fig. 2) but those for the Schiff-base exchange reaction were conducted in positive mode (Fig. 3a); the other positive or negative mode are shown in the Supporting Information (Fig. S3,a,c). We also attempted to dissolve crystal **2** in methanol, and high-intensity peaks for the [Dy(**L2**)_2_(solv.)] molecular fragment were found (Fig. S4,b).

Interestingly, the **L1** we used was not found in the structure, but 2-aminomethylpyridine replaced the position of taurine *in situ* with *o*-vanillin to form a Schiff base ligand. To investigate how 2-aminomethylpyridine replaced the 2-aminoethanesulfonic acid in **L1**, we performed a control experiment. **L1** + Dy(NO_3_)_3_·6H_2_O (without added 2-aminomethylpyridine) was reacted in methanol at room temperature for 24 h. The main fragment obtained through ESI-MS test reaction solution was **7** ([**L1]**^−^), and a large number of fragments **8** ([Dy(NO_3_)_4_]^−^) and **9** ([Dy(**L1**)(NO_3_)_2_]^−^) were also present. **L1** + 2-aminomethylpyridine (without added Dy(NO_3_)_3_·6H_2_O) was reacted in methanol at room temperature for 24 h, and the main fragment obtained with ESI-MS test reaction solution was 10 (7, [**L1**]^−^) (Figs 2 and S3). The above control experiments showed that the Schiff base replacement reaction did not occur without Ln(III) induction, i.e., 2-aminoethanesulfonic acid was first coordinated with Dy(III) ions in solution and then replaced with 2-aminomethylpyridine. Thus, the time-dependent tracking of the replacement process revealing its mechanism of replacement self-assembly was interesting. Notably, time-dependent assembly occurred at room temperature, which benefited slowing down the reaction rate and observing the process. It was followed by a 60 min time gradient. When **L1**, 2-aminomethylpyridine, and Dy(NO_3_)_3_·6H_2_O were added, no reaction occurred. About 1 min later, substitution occurred rapidly and the ligand after *in situ* replacement appeared at *m/z* = 243.11. The molecular formula was [(**L2**) + H]^+^ (*calc*. 243.11) by fitting, but **L1** ligand was reduced to 0.437 until it almost disappeared 3.5 min later. Moreover, with increased reaction time, the peak intensity of the **L2** fragment gradually decreased to 60 min. Two weak peaks appeared at *m/z* = 715.13 and 646.12, and [Dy(**L1**)(NO_3_)(solv.)]^+^ (*calc*. 715.13) and [Dy(**L2**)_2_]^+^ (*calc*.646.12) was obtained by fitting. However, with increased reaction time, the peak intensities of the two groups gradually increased. Figure 3 shows that the frame fragment of 646.12 obviously did not grow rapidly with 715.13. After 8 min, [Dy(**L1**)(NO_3_)(solv.)]^+^ gradually increased to the highest. However, after 45 min, the peak intensity of the molecular fragment of [Dy(**L2**)_2_]^+^ gradually increased to the highest until 60 min when the [Dy(**L1**)(NO_3_)(solv.)]^+^ framework fragment disappeared. Thus, driven by Dy(NO_3_)_3_·6H_2_O, 2-aminomethylpyridine gradually replaced taurine in **L1** with increased time and then assembled into the **2** frame (Figs S14, S15, and Table S12). In summary, through ESI-MS analysis, we speculated that the possible formation mechanism of **2** was [**L1**] → Dy[**L1**]/[**L2**] → Dy[**L2**] → Dy[**L2**]_2_ → Dy_2_[**L2**]_2_. The exchange of Schiff base occurred during the reaction in Dy[**L1**] → Dy[**L2**]. In this work, the Schiff base replacement reaction, which accompanied the assembly of the lanthanide complex, was studied through a combination of crystallography and mass spectrometry. Moreover, the most suitable time and stage of Schiff base replacement were clearly explained.

**Figure 4:**
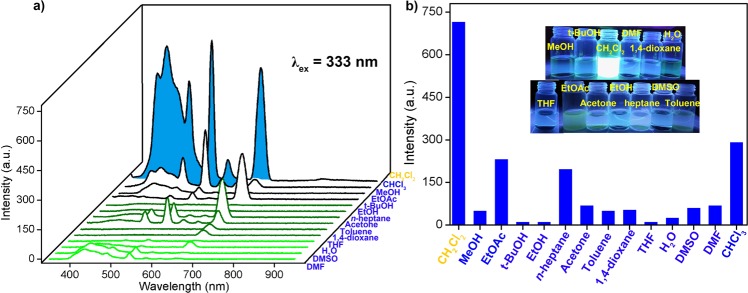
(**a**) Emission spectra of 1 dispersed in different organic molecules (5 mg/10 mL; excited at 333 nm). (**b**) Luminescence intensity of **1** with different organic molecules. The inset shows the corresponding photographs under UV-light irradiation at 365 nm.

Dichloromethane is the most common organic solvent and is widely used in organic synthesis. Its rapid and easy detection is a huge challenge. Based on the above luminescence studies (Figs S16 and 17), we used **1** as a fluorescent probe to examine its potential for sensing organic molecules. The as-obtained products (5 mg) were ground and dissolved in 10 mL of various organic solvents (CHCl_3_, MeOH, t-BuOH, CH_2_Cl_2_, DMF, 1,4-dioxane, H_2_O, THF, EtOAc, acetone, EtOH, *n*-heptane, DMSO, and toluene). When we excited **1** in different organic solvents at an excitation wavelength of 333 nm, we found that the emission peak of **1** dissolved in CH_2_Cl_2_ was remarkably higher than those of the other organic solvents. When **1** was dissolved and dispersed in CH_2_Cl_2_, it showed a strong, broad emission peak at 435 nm and strong emission peaks at 545 and 667 nm. Weak emission peaks were also observed at 490, 585, and 620 nm. When **1** was dissolved and dispersed in CHCl_3_, although it exhibited the same emission peak position as CH_2_Cl_2_, the luminescence intensity was weak. These emission peaks can be assigned to the π–π* level transition of the organic ligand **L2**, and the energy-level transition of the organic ligand (organic molecule) to the Tb(III) ion (Fig. 4a). To resolve CH_2_Cl_2_ quickly and easily, we placed a solution containing different organic molecules of **1** in a portable UV lamp at 365 nm. The fluorescence in the CH_2_Cl_2_ solution was clearly visible to the naked eye, whereas the emission in other organic solvents was weak. Therefore, the CH_2_Cl_2_ solution was quickly and easily distinguished (Fig. 4b).

**Figure 5:**
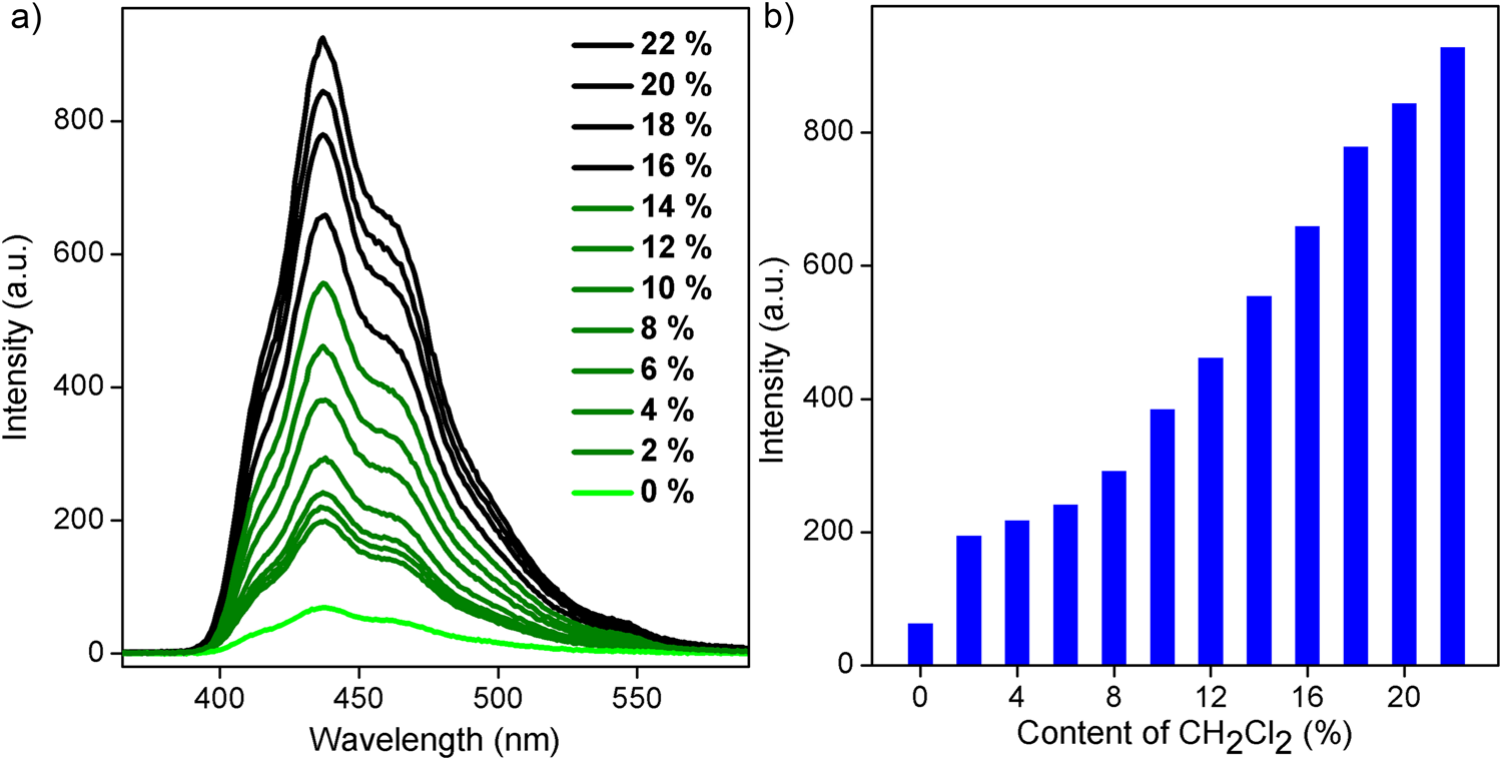
(**a**) Luminescence spectra of 1 dispersed in DMF with increased CH_2_Cl_2_ concentration (CH_2_Cl_2_/DMF). (**b**) Changes in fluorescence intensity with increased CH_2_Cl_2_ concentration in DMF solution.

We subsequently performed a luminescence titration experiment to examine the probe sensitivity. A certain amount of CH_2_Cl_2_ was separately added to the DMF solution containing **1** to test its fluorescence emission (Fig. 5a). Without CH_2_Cl_2_, the solution showed a weak luminescence with a strength of only 68, whereas the addition of 2% CH_2_Cl_2_ solution led to a remarkable increase in luminescence intensity to 198. With gradually increased CH_2_Cl_2_ content, the solution fluorescence gradually increased. With increased CH_2_Cl_2_ content to 22%, the luminous intensity of the solution increased to 928 (Fig. 5b). To verify the detection effect of **1** fluorescent probe on CH_2_Cl_2_ in sewage, we mixed a certain amount of CH_2_Cl_2_ and H_2_O, and the fluorescence increased rapidly with increased CH_2_Cl_2_ content in water (Fig. S18).

### Magnetic properties

DC magnetic susceptibilitiy studies of **1**, **2**, **3**, and **4** were conducted in a 1000 Oe field from 300 to 2 K (Fig. S19). At room temperature, the *χ*_M_*T* values of 23.75, 28.51, 28.22, and 23.13 cm^3^ K mol^−1^ were consistent with the expected values for two isolated Tb(III), Dy(III), Ho(III), and Er(III) ions, respectively. These *χ*_M_*T* values gradually decreased with decreased temperature until 50 K before decreasing rapidly to reach 9.48, 8.92, 7.61 and 9.13 cm^3^ K mol^−1^ at 2 K, respectively. The decreases may be attributed to the thermal depopulation of the Ln(III) Stark sublevels^29–33^. The maximum values of magnetization for **1**, **2**, **3**, and **4** were not saturated at 5 T and 2 K (Fig. S20). The saturation was not reached because the orbital contribution to the magnetic moment was very important and led to strong magnetic anisotropy. Alternating current (AC) magnetic susceptibilities suggest that only **2** exhibited weak frequency-dependent behavior in zero DC field (Fig. S21). Usually, a small magnetic field is efficient to observe the slow relaxation of magnetization and suppress the possible quantum tunneling effect of magnetization. Only a more obvious frequency-dependent behavior at 2000 Oe DC field, i.e., the effect of dc field on magnetic relaxation, was observed^34,35^. The Cole–Cole plots fitted by Debye model are shown in Fig. S22. The fitting parameters *τ* and *α* are listed in Tables S13. The parameter *α* ranged within 0.24–0.34, which showed the relatively moderate distribution of each relaxation time. Afterwards, we analyzed the temperature-dependent relaxation time by Arrhenius law (*τ* = *τ*_0_exp(*U*_eff_/*k*_B_*T*)), and the energy barrier and relaxation time were found to be *U*_eff1_ = 17.9 K and *τ*_1_ = 3.5 × 10^−7^ s under a 2000 Oe dc field (Fig. S22 and Table S13). An *S*-shaped magnetization hysteresis for **2** (Fig. S23) was observed with a steep rise at the crossing field *H* = 5 T.

## Conclusions

A rare *in situ* Schiff base exchange reaction was found and its process was examined by crystallography and mass spectrometry. The reaction was accompanied by a multistep assembly of lanthanide metal ions to afford a series of lanthanide-metal-complex fragment. The exchange of the Schiff base occurred after the original ligand **L1** was coordinated and then multistep assembly occurred to give the product, i.e., [**L1**] → Dy[**L1**]/[**L2**] → Dy[**L2**] → Dy[**L2**]_2_ → Dy_2_[**L2**]_2_. Furthermore, the quick, easy, and selective identification of CH_2_Cl_2_ was realized using **1** as fluorescence probe. To the best of our knowledge, this study is the first to explore the quick and easy fluorescence sensing of CH_2_Cl_2_. This work shows the great potential of lanthanide metal ions for the *in situ* Schiff base exchange and as a fluorescence probe. Our research method described an *in situ* reaction during complex self-assembly, which may provide new insights into the quick and easy detection of CH_2_Cl_2_ and thus the foundation for the discovery of highly effective and convenient detection methods.

## Experimental Section

### Materials and measurements

All reagents were obtained from commercial sources and used without further purification. Elemental (C, H, and N) analysis was conducted on an Elementar Micro cube elemental analyzer. Thermal analysis was performed in N_2_ at a heating rate of 5 °C/min using Labsys Evo TG-DTG/DSC. IR spectra with KBr pellet were recorded on PE Spectrum Two FT/IR spectrometer (400–4000 cm^−1^). PXRD measurements were recorded on a Rigaku D/max-IIIA diffractometer. Magnetic susceptibility was measured with a MPMS SQUID-XL magnetometer equipped with 5 T magnet within the temperature range of 2–300 K. Diamagnetic corrections were estimated using Pascal’s constants. AC susceptibility was measured and data were collected at increasing temperatures from 2 K to 10 K within frequencies ranging from 1 Hz to 1000 Hz and a drive frequency of 2.5 Oe.

### Single-crystal X-ray crystallography

The diffraction data of all complexes were obtained on a Bruker SMART CCD diffractometer (Mo Kα radiation and *λ* = 0.71073 Å) in *Φ* and *ω* scan modes. All structures were solved by direct methods followed by difference Fourier syntheses and then refined by full-matrix least-square techniques on *F*^2^ using SHELXL^36^. All other non-hydrogen atoms were refined with anisotropic thermal parameters. Hydrogen atoms were placed in the calculated position and refined in the isotropic direction using a riding model. Table S1 summarizes X-ray crystallographic data and refinement details of the complexes. Complete details can be found in the CIF files provided in the Supporting Information. The CCDC reference numbers are 1879618 for **1**, 1879615 for **2**, 1879617 for **3**, and 1879616 for **4**.

### High-resolution ESI-MS test

High-resolution ESI-MS were performed at the capillary temperature of 275 °C, and the solution was injected at a rate of 0.3 mL/h. The ESI-MS used for the measurements was a ThermoExactive, and data were collected in positive and negative ion modes. The spectrometer was previously calibrated with the standard tune mix to give a precision of *ca*. 2 ppm within the range of 200–2500 *m/z*. The capillary voltage was 50 V, the tube lens voltage was 150 V, and the skimmer voltage was 25 V. The in-source energy was set within the range of 0–100 eV with a gas flow rate at 10% of the maximum.

### Synthesis of complexes 1, 2, 3, and 4

The mixture of [Li_2_**L1**] ligand (0.5 mmol), Ln(NO_3_)_3_·6H_2_O (0.5 mmol), 2-aminomethylpyridine (2 mmol), and 10 mL CH_3_OH were stirred for 2 h in 25 mL Bunsen beaker and then volatilized at room temperature for 12 h to obtain yellow crystals. **1**: yield: 75% (based on Tb(NO_3_)_3_·6H_2_O). IR data for (KBr, cm^−1^): 3411 (*m*), 1645 (*m*), 1500 (*s*), 1430 (*s*), 1384 (*s*), 1272 (*s*), 1038 (*m*), 893 (*s*), 813 (*m*), 742 (*s*), 645 (*w*), 523 (*m*), 492 (*s*), 434 (*w*). Elemental analyses *calc* (%) for [Tb_2_(**L2**)_2_(NO_3_)_4_]·2CH_3_OH: C 32.56, H 2.55, N 10.13; Found: C 32.39, H 2.67, N 10.06. **2:** yield: 72% (based on Dy(NO_3_)_3_·6H_2_O). IR data (KBr, cm^−1^): 3410 (*m*), 1644 (*m*), 1509 (*s*), 1461 (*s*), 1384 (*s*), 1272 (*s*), 1037 (*m*), 892 (*s*), 813 (*m*), 745 (*s*), 642 (*w*), 522 (*m*), 495 (*s*), 430 (*w*). Elemental analyses *calc* (%) for [Dy_2_(**L2**)_2_(NO_3_)_4_]·2CH_3_OH: C 32.36, H 2.53, N 10.06; Found: C 31.21, H 2.69, N 10.01. **3**: yield: 77% (based on Ho(NO_3_)_3_·6H_2_O). IR data (KBr, cm^−1^): 3405 (*m*), 1646 (*m*), 1501 (*s*), 1431 (*s*), 1384 (*s*), 1273 (*s*), 1039 (*m*), 893 (*s*), 813 (*m*), 743 (*s*), 641 (*w*), 525 (*m*), 492 (*s*), 438 (*w*). Elemental analyses *calc* (%) for [Ho_2_(**L2**)_2_(NO_3_)_4_]·2CH_3_OH: C 32.22, H 2.52, N 10.02; Found: C 32.05, H 2.63, N 9.98. **4:**yield: 71% (based on Er(NO_3_)_3_·6H_2_O). IR data (KBr, cm^−1^): 3400 (*m*), 1646 (*m*), 1503 (*s*), 1431 (*s*), 1384 (*s*), 1276 (*s*), 1039 (*m*), 898 (*s*), 815 (*m*), 742 (*s*), 643 (*w*), 522 (*m*), 490 (*s*), 434 (*w*). Elemental analyses *calc* (%) for [Er_2_(**L2**)_2_(NO_3_)_4_]·2CH_3_OH: C 32.08, H 2.51, N 9.98; Found: C 31.99, H 2.58, N 9.93.

### Synthesis of ligand HL2

O-vanillin (1 mmol) was dissolved in 25 mL of anhydrous methanol and then 2-aminomethylpyridine (1 mmol) was added. The mixture was stirred overnight and methanol was evaporated to give a yellow solid. Yield: 84% (based on o-vanillin). ([HL2 + H]^+^ *Expt*.*m/z* = 243.11, *Calc*. *m/z* = 243.10.

## Supplementary information

Supplementary information

Supplementary information

Supplementary information
