# Supplementary material for: Tracking the Multistep Formation of Ln(III) Complexes with in situ Schiff Base Exchange Reaction and its Highly Selective Sensing of Dichloromethane
Source: Sci Rep. 2019 Aug 22;9:12231. doi: 10.1038/s41598-019-48696-y (PMC6706376; doi:10.1038/s41598-019-48696-y)

Tracking the Multistep Formation of Ln(III) Complexes with *in situ* Schiff Base Exchange Reaction and its Highly Selective Sensing of Dichloromethane

Kai-Qiang Mo, [1,+] Xiong-Feng Ma, [1,+] Hai-Ling Wang, [1,+] Zhong-Hong Zhu,*, [1] Yan-Cheng Liu, [1] Hua-Hong Zou,*, [1] Fu-Pei Liang,*, [1]

[1]State Key Laboratory for Chemistry and Molecular Engineering of Medicinal Resources, School of Chemistry & Pharmacy of Guangxi Normal University, Guilin 541004, P. R. China. E-mail: gxnuchem@foxmail.com, 18317725515@163.com, fliangoffice@yahoo.com.

checkCIF/PLATON report

You have not supplied any structure factors. As a result the full set of tests cannot be run.

THIS REPORT IS FOR GUIDANCE ONLY. IF USED AS PART OF A REVIEW PROCEDURE FOR PUBLICATION, IT SHOULD NOT REPLACE THE EXPERTISE OF AN EXPERIENCED CRYSTALLOGRAPHIC REFEREE.

No syntax errors found. CIF dictionary Interpreting this report

Datablock: 183_tbaaaa

```
Bond precision:  C-C = 0.0066 A          Wavelength=0.71073

Cell:           a=9.9763(2)       b=10.0494(2)      c=11.1816(3)
                alpha=67.201(2)   beta=72.780(2)    gamma=87.564(2)
Temperature:    293 K
```

	Calculated	Reported
Volume	983.79(4)	983.79(4)
Space group	P -1	P -1
Hall group	-P 1	-P 1
Moiety formula	C28 H26 N8 O16 Tb2, 2(C H O)	C28 H26 N8 O16 Tb2, 2(C H O)
Sum formula	C30 H28 N8 O18 Tb2	C30 H28 N8 O18 Tb2
Mr	1106.46	1106.44
Dx,g cm-3	1.868	1.868
Z	1	1
Mu (mm-1)	3.650	3.650
F000	538.0	538.0
F000’	537.92	
h,k,lmax	12,12,13	12,12,13
Nref	3660	3652
Tmin,Tmax		0.622,1.000
Tmin’		

```
Correction method= # Reported T Limits: Tmin=0.622 Tmax=1.000
AbsCorr = MULTI-SCAN

Data completeness= 0.998          Theta(max)= 25.500
```

R(reflections)= 0.0220(3406)   wR2(reflections)= 0.0587(3652)

S = 1.104   Npar= 272

```
The following ALERTS were generated. Each ALERT has the format
       test-name_ALERT_alert-type_alert-level.
Click on the hyperlinks for more details of the test.
```

Alert level C

```
PLAT053_ALERT_1_C Minimum Crystal Dimension Missing (or Error) ...     Please Check
PLAT054_ALERT_1_C Medium Crystal Dimension Missing (or Error) ...      Please Check
PLAT055_ALERT_1_C Maximum Crystal Dimension Missing (or Error) ...     Please Check
PLAT241_ALERT_2_C High 'MainMol' Ueq as Compared to Neighbors of       O6 Check
PLAT242_ALERT_2_C Low 'MainMol' Ueq as Compared to Neighbors of        N4 Check
PLAT260_ALERT_2_C Large Average Ueq of Residue Including       O9     0.126 Check
```

Alert level G

```
PLAT003_ALERT_2_G Number of Uiso or Uij Restrained non-H Atoms ...         24 Report
PLAT005_ALERT_5_G No Embedded Refinement Details Foundin the CIF Please Do !
PLAT154_ALERT_1_G The s.u.'s on the Cell Angles are Equal ..(Note)      0.002 Degree
PLAT199_ALERT_1_G Reported _cell_measurement_temperature ..... (K)        293 Check
PLAT200_ALERT_1_G Reported   _diffrn_ambient_temperature ..... (K)        293 Check
PLAT232_ALERT_2_G Hirshfeld Test Diff (M-X)  Tb1      --O6       .        5.3 s.u.
PLAT232_ALERT_2_G Hirshfeld Test Diff (M-X)  Tb1      --O7       .        6.6 s.u.
PLAT300_ALERT_4_G Atom Site Occupancy of C0AAConstrained at      0.5 Check
PLAT300_ALERT_4_G Atom Site Occupancy of C15        Constrained at        0.5 Check
PLAT300_ALERT_4_G Atom Site Occupancy of H15AConstrained at      0.5 Check
PLAT300_ALERT_4_G Atom Site Occupancy of H15BConstrained at      0.5 Check
PLAT302_ALERT_4_G Anion/Solvent/Minor-Residue Disorder (Resd  2  )        50% Note
PLAT720_ALERT_4_G Number of Unusual/Non-Standard Labels ..........          1 Note
PLAT764_ALERT_4_G Overcomplete CIF Bond List Detected (Rep/Expd) .       1.14 Ratio
PLAT794_ALERT_5_G Tentative Bond Valency for Tb1       (III)     .       3.63 Info
PLAT860_ALERT_3_G Number of Least-Squares Restraints ............         294 Note
```

```
   0 ALERT level A = Most likely a serious problem - resolve or explain
   0 ALERT level B = A potentially serious problem, consider carefully
   6 ALERT level C = Check. Ensure it is not caused by an omission or oversight
  16 ALERT level G = General information/check it is not something unexpected

   6 ALERT type 1 CIF construction/syntax error, inconsistent or missing data
   6 ALERT type 2 Indicator that the structure model may be wrong or deficient
   1 ALERT type 3 Indicator that the structure quality may be low
   7 ALERT type 4 Improvement, methodology, query or suggestion
   2 ALERT type 5 Informative message, check
```

Datablock: 183-dyaaaa

```
Bond precision:  C-C = 0.0072 A          Wavelength=0.71073

Cell:           a=9.9718(4)        b=10.0164(3)       c=11.1731(5)
                alpha=67.350(4)    beta=72.714(4)     gamma=87.627(3)
Temperature:    293 K
```

	Calculated	Reported
Volume	980.02(8)	980.02(7)
Space group	P -1	P -1
Hall group	-P 1	-P 1
Moiety formula	C28 H26 Dy2 N8 O16, 2(C H O)	C28 H26 Dy2 N8 O16, 2(C H O)
Sum formula	C30 H28 Dy2 N8 O18	C30 H28 Dy2 N8 O18
Mr	1113.60	1113.60
Dx,g cm-3	1.887	1.887
Z	1	1
Mu (mm-1)	3.868	3.868
F000	540.0	540.0
F000’	539.89	
h,k,lmax	12,12,13	12,12,13
Nref	3650	3642
Tmin,Tmax		0.688,1.000
Tmin’		

Correction method= # Reported T Limits: Tmin=0.688 Tmax=1.000
AbsCorr = MULTI-SCAN

Data completeness= 0.998 Theta(max)= 25.500

R(reflections)= 0.0228(3366) wR2(reflections)= 0.0603(3642)

S = 1.094 Npar= 272

The following ALERTS were generated. Each ALERT has the format
test-name_ALERT_alert-type_alert-level.
Click on the hyperlinks for more details of the test.

Alert level C

```
PLAT053_ALERT_1_C Minimum Crystal Dimension Missing (or Error) ...     Please Check
PLAT054_ALERT_1_C Medium Crystal Dimension Missing (or Error) ...     Please Check
PLAT055_ALERT_1_C Maximum Crystal Dimension Missing (or Error) ...    Please Check
PLAT241_ALERT_2_C High 'MainMol' Ueq as Compared to Neighbors of  O7   Check
PLAT242_ALERT_2_C Low 'MainMol' Ueq as Compared to Neighbors of  N4    Check
PLAT260_ALERT_2_C Large Average Ueq of Residue Including       O9      0.131 Check
```

Alert level G

```
PLAT005_ALERT_5_G No Embedded Refinement Details Found  in the CIF     Please Do !
PLAT199_ALERT_1_G Reported _cell_measurement_temperature ..... (K)        293 Check
PLAT200_ALERT_1_G Reported   _diffrn_ambient_temperature ..... (K)        293 Check
PLAT232_ALERT_2_G Hirshfeld Test Diff (M-X)  Dy1      --O6       .        6.2 s.u.
PLAT232_ALERT_2_G Hirshfeld Test Diff (M-X)  Dy1      --O7       .        5.2 s.u.
PLAT300_ALERT_4_G Atom Site Occupancy of C0AA       Constrained at        0.5 Check
PLAT300_ALERT_4_G Atom Site Occupancy of C15        Constrained at        0.5 Check
PLAT300_ALERT_4_G Atom Site Occupancy of H15A       Constrained at        0.5 Check
PLAT300_ALERT_4_G Atom Site Occupancy of H15B       Constrained at        0.5 Check
PLAT302_ALERT_4_G Anion/Solvent/Minor-Residue Disorder (Resd  2  )        50% Note
PLAT720_ALERT_4_G Number of Unusual/Non-Standard Labels .........           1 Note
```

```
PLAT764_ALERT_4_G Overcomplete CIF Bond List Detected (Rep/Expd) .       1.14 Ratio
PLAT794_ALERT_5_G Tentative Bond Valency for Dy1       (II)      .       1.73 Info
```

```
   0 ALERT level A = Most likely a serious problem - resolve or explain
   0 ALERT level B = A potentially serious problem, consider carefully
   6 ALERT level C = Check. Ensure it is not caused by an omission or oversight
  13 ALERT level G = General information/check it is not something unexpected

   5 ALERT type 1 CIF construction/syntax error, inconsistent or missing data
   5 ALERT type 2 Indicator that the structure model may be wrong or deficient
   0 ALERT type 3 Indicator that the structure quality may be low
   7 ALERT type 4 Improvement, methodology, query or suggestion
   2 ALERT type 5 Informative message, check
```

Datablock: 183-hoaaaa

Bond precision: C-C = 0.0072 A   Wavelength=0.71073

Cell:	a=9.9472(4)	b=9.9963(3)	c=11.1721(5)
	alpha=67.388(4)	beta=72.530(3)	gamma=87.401(3)
Temperature:	293 K		

	Calculated	Reported
Volume	975.10(7)	975.10(7)
Space group	P -1	P -1
Hall group	-P 1	-P 1
Moiety formula	C28 H26 Ho2 N8 O16, 2(C H O)	C28 H26 Ho2 N8 O16, 2(C H O)
Sum formula	C30 H28 Ho2 N8 O18	C30 H28 Ho2 N8 O18
Mr	1118.46	1118.46
Dx,g cm-3	1.905	1.905
Z	1	1
Mu (mm-1)	4.113	4.113
F000	542.0	542.0
F000’	541.84	
h,k,lmax	12,12,13	12,12,13
Nref	3629	3623
Tmin,Tmax		0.360,1.000
Tmin’		

Correction method= # Reported T Limits: Tmin=0.360 Tmax=1.000
AbsCorr = MULTI-SCAN

Data completeness= 0.998   Theta(max)= 25.490

R(reflections)= 0.0230(3393)   wR2(reflections)= 0.0603(3623)

```
S = 1.075                    Npar= 272
```

The following ALERTS were generated. Each ALERT has the format
       test-name_ALERT_alert-type_alert-level.
Click on the hyperlinks for more details of the test.

Alert level C

```
PLAT053_ALERT_1_C Minimum Crystal Dimension Missing (or Error) ...     Please Check
PLAT054_ALERT_1_C Medium Crystal Dimension Missing (or Error) ...  Please Check
PLAT055_ALERT_1_C Maximum Crystal Dimension Missing (or Error) ...  Please Check
PLAT241_ALERT_2_C High 'MainMol' Ueq as Compared to Neighbors of O6 Check
PLAT242_ALERT_2_C Low 'MainMol' Ueq as Compared to Neighbors of N4 Check
PLAT260_ALERT_2_C Large Average Ueq of Residue Including        O9      0.128 Check
```

Alert level G

```
PLAT005_ALERT_5_G No Embedded Refinement Details Found  in the CIF     Please Do !
PLAT199_ALERT_1_G Reported _cell_measurement_temperature ..... (K)        293 Check
PLAT200_ALERT_1_G Reported   _diffrn_ambient_temperature ..... (K)        293 Check
PLAT232_ALERT_2_G Hirshfeld Test Diff (M-X)  Ho1      --O6       .        5.3 s.u.
PLAT232_ALERT_2_G Hirshfeld Test Diff (M-X)  Ho1      --O7       .        5.8 s.u.
PLAT300_ALERT_4_G Atom Site Occupancy of C0AA       Constrained at        0.5 Check
PLAT300_ALERT_4_G Atom Site Occupancy of C15        Constrained at        0.5 Check
PLAT300_ALERT_4_G Atom Site Occupancy of H15A       Constrained at        0.5 Check
PLAT300_ALERT_4_G Atom Site Occupancy of H15B       Constrained at        0.5 Check
PLAT302_ALERT_4_G Anion/Solvent/Minor-Residue Disorder (Resd  2  )        50% Note
PLAT710_ALERT_4_G Delete 1-2-3 or 2-3-4 Linear Torsion Angle ... #          8 Do !
              HO1 -HO1 -N3  -O5   -139.00 15.00   2.656   1.555   1.555   1.555
PLAT710_ALERT_4_G Delete 1-2-3 or 2-3-4 Linear Torsion Angle ... #         10 Do !
              HO1 -HO1 -N4  -O8   -111.00  5.00   2.656   1.555   1.555   1.555
PLAT710_ALERT_4_G Delete 1-2-3 or 2-3-4 Linear Torsion Angle ... #         49 Do !
              O2  -HO1 -N3  -O5   -102.00 15.00   2.656   1.555   1.555   1.555
PLAT710_ALERT_4_G Delete 1-2-3 or 2-3-4 Linear Torsion Angle ... #         50 Do !
              O2  -HO1 -N3  -O5   -176.00100.00   1.555   1.555   1.555   1.555
PLAT710_ALERT_4_G Delete 1-2-3 or 2-3-4 Linear Torsion Angle ... #         53 Do !
              O2  -HO1 -N4  -O8   -148.00  5.00   2.656   1.555   1.555   1.555
PLAT710_ALERT_4_G Delete 1-2-3 or 2-3-4 Linear Torsion Angle ... #         54 Do !
              O2  -HO1 -N4  -O8    -74.00  5.00   1.555   1.555   1.555   1.555
PLAT710_ALERT_4_G Delete 1-2-3 or 2-3-4 Linear Torsion Angle ... #         75 Do !
              O1  -HO1 -N3  -O5    -36.00 15.00   2.656   1.555   1.555   1.555
PLAT710_ALERT_4_G Delete 1-2-3 or 2-3-4 Linear Torsion Angle ... #         77 Do !
              O1  -HO1 -N4  -O8    147.00  5.00   2.656   1.555   1.555   1.555
PLAT710_ALERT_4_G Delete 1-2-3 or 2-3-4 Linear Torsion Angle ... #         92 Do !
              O4  -HO1 -N3  -O5    -48.00 15.00   1.555   1.555   1.555   1.555
PLAT710_ALERT_4_G Delete 1-2-3 or 2-3-4 Linear Torsion Angle ... #         94 Do !
              O4  -HO1 -N4  -O8    141.00  5.00   1.555   1.555   1.555   1.555
PLAT710_ALERT_4_G Delete 1-2-3 or 2-3-4 Linear Torsion Angle ... #        106 Do !
              O3  -HO1 -N3  -O5    133.00 15.00   1.555   1.555   1.555   1.555
PLAT710_ALERT_4_G Delete 1-2-3 or 2-3-4 Linear Torsion Angle ... #        108 Do !
              O3  -HO1 -N4  -O8     -7.00  5.00   1.555   1.555   1.555   1.555
PLAT710_ALERT_4_G Delete 1-2-3 or 2-3-4 Linear Torsion Angle ... #        121 Do !
              O7  -HO1 -N3  -O5     36.00 15.00   1.555   1.555   1.555   1.555
PLAT710_ALERT_4_G Delete 1-2-3 or 2-3-4 Linear Torsion Angle ... #        122 Do !
              O7  -HO1 -N4  -O8     80.00  5.00   1.555   1.555   1.555   1.555
PLAT710_ALERT_4_G Delete 1-2-3 or 2-3-4 Linear Torsion Angle ... #        135 Do !
              O6  -HO1 -N3  -O5   -153.00 15.00   1.555   1.555   1.555   1.555
PLAT710_ALERT_4_G Delete 1-2-3 or 2-3-4 Linear Torsion Angle ... #        137 Do !
              O6  -HO1 -N4  -O8   -105.00  5.00   1.555   1.555   1.555   1.555
PLAT710_ALERT_4_G Delete 1-2-3 or 2-3-4 Linear Torsion Angle ... #        146 Do !
```

```
              N3  -HO1 -N4  -      -22.40  0.80   1.555   1.555   1.555   1.555
PLAT710_ALERT_4_G Delete 1-2-3 or 2-3-4 Linear Torsion Angle ... #        147 Do !
              N3  -HO1 -N4  -       57.00  5.00   1.555   1.555   1.555   1.555
PLAT710_ALERT_4_G Delete 1-2-3 or 2-3-4 Linear Torsion Angle ... #        148 Do !
              N3  -HO1 -N4  -      161.90  0.60   1.555   1.555   1.555   1.555
PLAT710_ALERT_4_G Delete 1-2-3 or 2-3-4 Linear Torsion Angle ... #        157 Do !
              N4  -HO1 -N3  -      101.20  0.70   1.555   1.555   1.555   1.555
PLAT710_ALERT_4_G Delete 1-2-3 or 2-3-4 Linear Torsion Angle ... #        158 Do !
              N4  -HO1 -N3  -      -79.70  0.70   1.555   1.555   1.555   1.555
PLAT710_ALERT_4_G Delete 1-2-3 or 2-3-4 Linear Torsion Angle ... #        159 Do !
              N4  -HO1 -N3  -       53.00 16.00   1.555   1.555   1.555   1.555
PLAT710_ALERT_4_G Delete 1-2-3 or 2-3-4 Linear Torsion Angle ... #        172 Do !
              N2  -HO1 -N3  -       43.00 15.00   1.555   1.555   1.555   1.555
PLAT710_ALERT_4_G Delete 1-2-3 or 2-3-4 Linear Torsion Angle ... #        174 Do !
              N2  -HO1 -N4  -       67.00  5.00   1.555   1.555   1.555   1.555
PLAT710_ALERT_4_G Delete 1-2-3 or 2-3-4 Linear Torsion Angle ... #        188 Do !
              N1  -HO1 -N3  -      106.00 15.00   1.555   1.555   1.555   1.555
PLAT710_ALERT_4_G Delete 1-2-3 or 2-3-4 Linear Torsion Angle ... #        190 Do !
              N1  -HO1 -N4  -        3.00  5.00   1.555   1.555   1.555   1.555
PLAT720_ALERT_4_G Number of Unusual/Non-Standard Labels .........           1 Note
PLAT764_ALERT_4_G Overcomplete CIF Bond List Detected (Rep/Expd) .       1.14 Ratio
PLAT794_ALERT_5_G Tentative Bond Valency for Ho1       (III)     .       2.50 Info
```

---

0 **ALERT level A** = Most likely a serious problem - resolve or explain
0 **ALERT level B** = A potentially serious problem, consider carefully
6 **ALERT level C** = Check. Ensure it is not caused by an omission or oversight
39 **ALERT level G** = General information/check it is not something unexpected

5 ALERT type 1 CIF construction/syntax error, inconsistent or missing data
5 ALERT type 2 Indicator that the structure model may be wrong or deficient
0 ALERT type 3 Indicator that the structure quality may be low
33 ALERT type 4 Improvement, methodology, query or suggestion
2 ALERT type 5 Informative message, check

Datablock: 183_eraaaa

Bond precision: C-C = 0.0083 A Wavelength=0.71073

Cell:	a=9.9436(5)	b=9.9985(9)	c=11.1634(8)
	alpha=67.488(8)	beta=72.552(6)	gamma=87.384(6)
Temperature:	293 K		

	Calculated	Reported
Volume	975.11(14)	975.12(12)
Space group	P -1	P -1
Hall group	-P 1	-P 1
Moiety formula	C28 H26 Er2 N8 O16, 2(C H O)	C28 H26 Er2 N8 O16, 2(C H O)
Sum formula	C30 H28 Er2 N8 O18	C30 H28 Er2 N8 O18
Mr	1123.12	1123.12
Dx,g cm-3	1.913	1.913
Z	1	1
Mu (mm-1)	4.359	4.359
F000	544.0	544.0
F000’	543.77	
h,k,lmax	12,12,13	12,12,13
Nref	3632	3624
Tmin,Tmax		0.516,1.000
Tmin’		

Correction method= # Reported T Limits: Tmin=0.516 Tmax=1.000
AbsCorr = MULTI-SCAN

Data completeness= 0.998 Theta(max)= 25.500

R(reflections)= 0.0285(3408) wR2(reflections)= 0.0758(3624)

S = 1.089 Npar= 272

The following ALERTS were generated. Each ALERT has the format
test-name_ALERT_alert-type_alert-level.
Click on the hyperlinks for more details of the test.

Alert level C

PLAT053_ALERT_1_C Minimum Crystal Dimension Missing (or Error) ... Please Check
PLAT054_ALERT_1_C Medium Crystal Dimension Missing (or Error) ... Please Check
PLAT055_ALERT_1_C Maximum Crystal Dimension Missing (or Error) ... Please Check
PLAT241_ALERT_2_C High 'MainMol' Ueq as Compared to Neighbors of O6 Check
PLAT260_ALERT_2_C Large Average Ueq of Residue Including O9 0.120 Check
PLAT342_ALERT_3_C Low Bond Precision on C-C Bonds .... 0.00833 Ang.

Alert level G

PLAT003_ALERT_2_G Number of Uiso or Uij Restrained non-H Atoms ... 2 Report
PLAT005_ALERT_5_G No Embedded Refinement Details Foundin the CIF Please Do !
PLAT152_ALERT_1_G The Supplied and Calc. Volume s.u. Differ by ... 2 Units
PLAT199_ALERT_1_G Reported _cell_measurement_temperature ..... (K) 293 Check
PLAT200_ALERT_1_G Reported _diffrn_ambient_temperature ..... (K) 293 Check
PLAT232_ALERT_2_G Hirshfeld Test Diff (M-X) Er1 --O6 . 5.5 s.u.
PLAT232_ALERT_2_G Hirshfeld Test Diff (M-X) Er1 --O7 . 6.0 s.u.
PLAT300_ALERT_4_G Atom Site Occupancy of C15 Constrained at 0.5 Check
PLAT300_ALERT_4_G Atom Site Occupancy of C16 Constrained at 0.5 Check
PLAT300_ALERT_4_G Atom Site Occupancy of H15A Constrained at 0.5 Check
PLAT300_ALERT_4_G Atom Site Occupancy of H15B Constrained at 0.5 Check

```
PLAT302_ALERT_4_G Anion/Solvent/Minor-Residue Disorder (Resd  2  )        50% Note
PLAT764_ALERT_4_G Overcomplete CIF Bond List Detected (Rep/Expd) .       1.14 Ratio
PLAT790_ALERT_4_G Centre of Gravity not Within Unit Cell: Resd.  #          2 Note
              C H O
PLAT794_ALERT_5_G Tentative Bond Valency for Er1       (II)      .       1.71 Info
PLAT860_ALERT_3_G Number of Least-Squares Restraints ............          12 Note
```

```
   0 ALERT level A = Most likely a serious problem - resolve or explain
   0 ALERT level B = A potentially serious problem, consider carefully
   6 ALERT level C = Check. Ensure it is not caused by an omission or oversight
  16 ALERT level G = General information/check it is not something unexpected

   6 ALERT type 1 CIF construction/syntax error, inconsistent or missing data
   5 ALERT type 2 Indicator that the structure model may be wrong or deficient
   2 ALERT type 3 Indicator that the structure quality may be low
   7 ALERT type 4 Improvement, methodology, query or suggestion
   2 ALERT type 5 Informative message, check
```

It is advisable to attempt to resolve as many as possible of the alerts in all categories. Often the minor alerts point to easily fixed oversights, errors and omissions in your CIF or refinement strategy, so attention to these fine details can be worthwhile. In order to resolve some of the more serious problems it may be necessary to carry out additional measurements or structure refinements. However, the purpose of your study may justify the reported deviations and the more serious of these should normally be commented upon in the discussion or experimental section of a paper or in the "special_details" fields of the CIF. checkCIF was carefully designed to identify outliers and unusual parameters, but every test has its limitations and alerts that are not important in a particular case may appear. Conversely, the absence of alerts does not guarantee there are no aspects of the results needing attention. It is up to the individual to critically assess their own results and, if necessary, seek expert advice.

Publication of your CIF in IUCr journals

A basic structural check has been run on your CIF. These basic checks will be run on all CIFs submitted for publication in IUCr journals (*Acta Crystallographica*, *Journal of Applied Crystallography*, *Journal of Synchrotron Radiation*); however, if you intend to submit to *Acta Crystallographica Section C* or *E* or *IUCrData*, you should make sure that full publication checks are run on the final version of your CIF prior to submission.

Publication of your CIF in other journals

Please refer to the *Notes for Authors* of the relevant journal for any special instructions relating to CIF submission.

PLATON version of 19/10/2018; check.def file version of 15/10/2018

Datablock 183_tbaaaa - ellipsoid plot

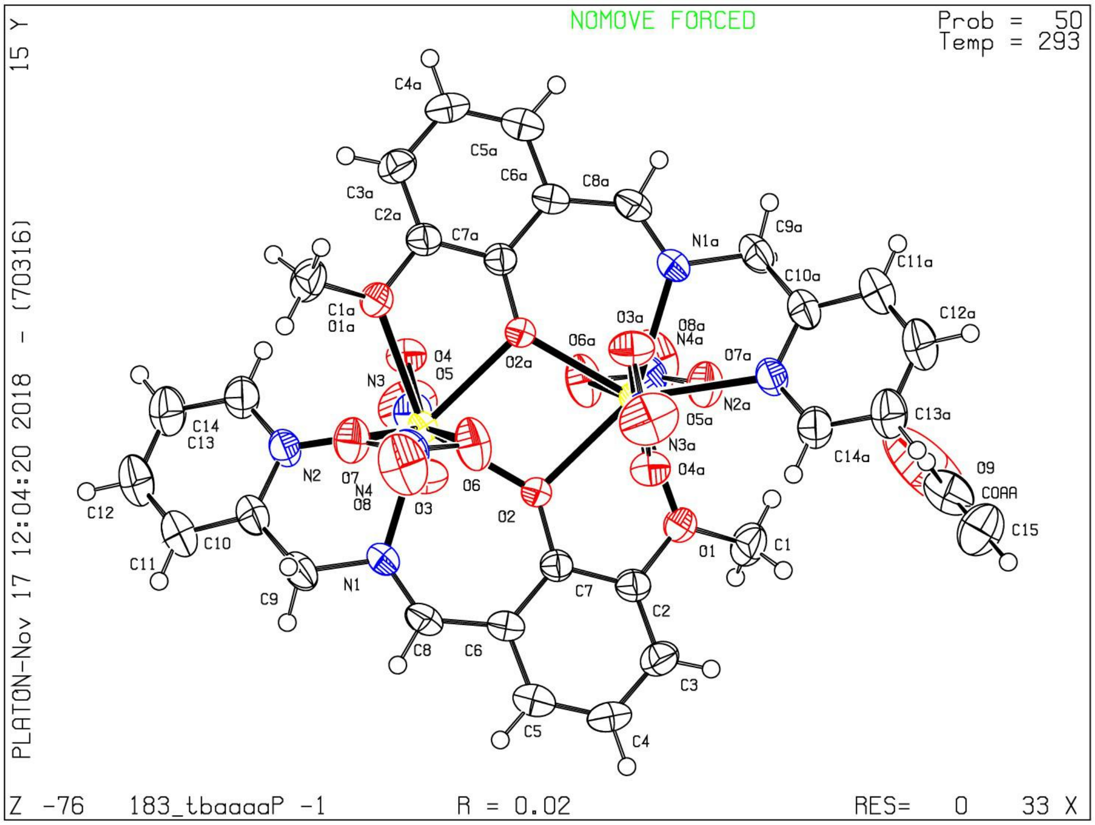

Datablock 183-dyaaaa - ellipsoid plot

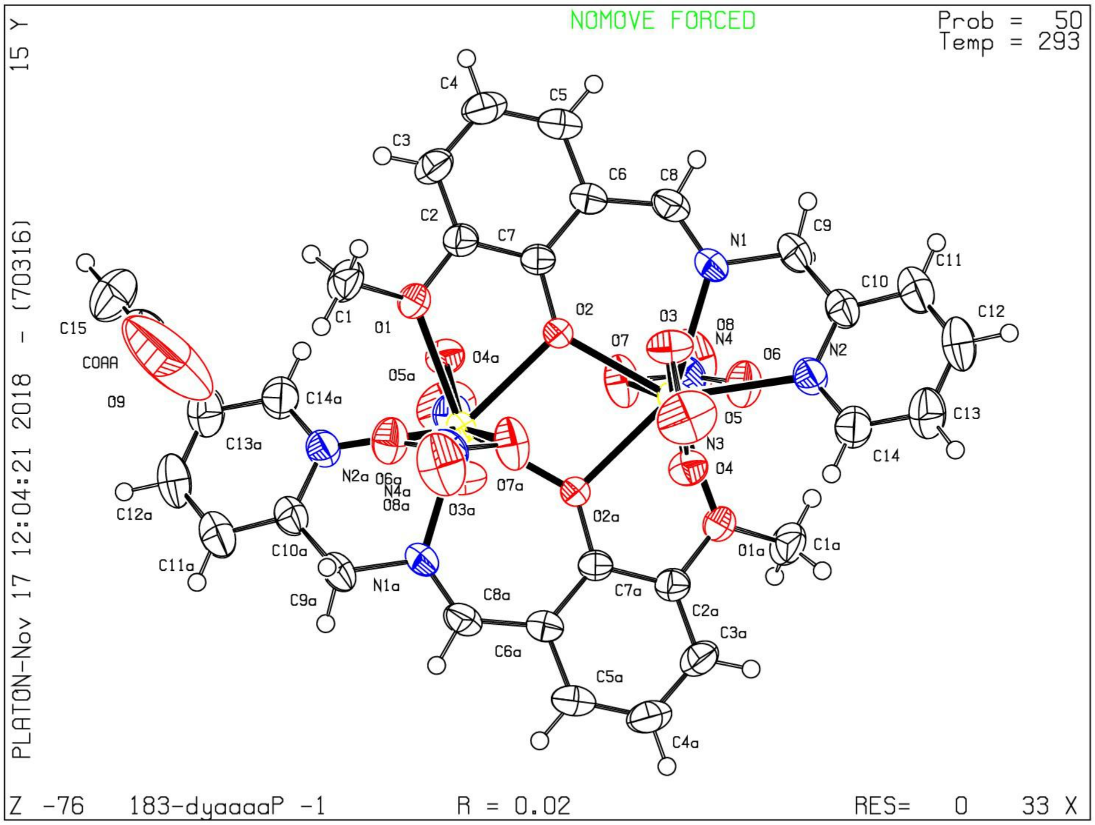

Datablock 183-hoaaaa - ellipsoid plot

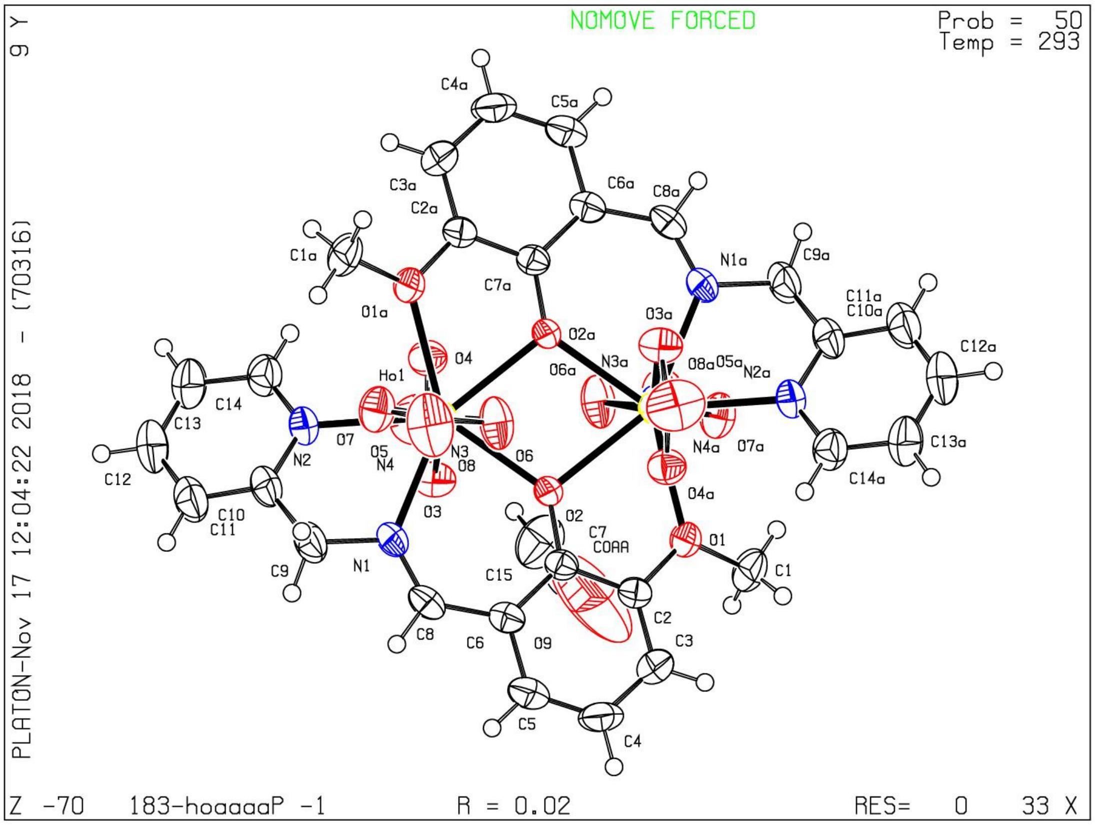

Datablock 183_eraaaa - ellipsoid plot

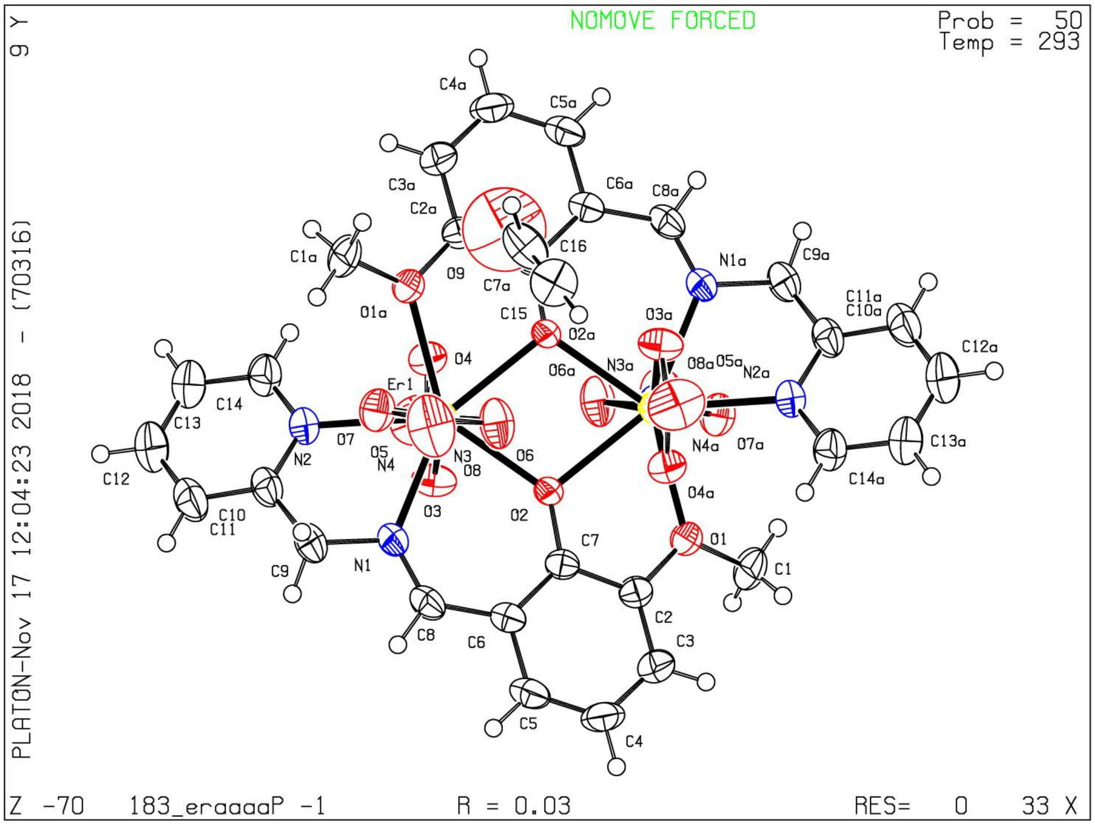

Supplement: Supplementary file 1 — Supplementary information [file 41598_2019_48696_MOESM1_ESM.pdf]
