# Supplementary material for: Tracking the Multistep Formation of Ln(III) Complexes with in situ Schiff Base Exchange Reaction and its Highly Selective Sensing of Dichloromethane
Source: Sci Rep. 2019 Aug 22;9:12231. doi: 10.1038/s41598-019-48696-y (PMC6706376; doi:10.1038/s41598-019-48696-y)
Supplement: Supplementary file 3 — Supplementary information [file 41598_2019_48696_MOESM3_ESM.docx]

*Supporting Information*

**Tracking the Multistep Formation of Ln(III) Complexes with *in situ* Schiff Base Exchange Reaction and its Highly Selective Sensing of Dichloromethane**

Kai-Qiang Mo, ^1,+^ Xiong-Feng Ma, ^1,+^ Hai-Ling Wang, ^1,+^ Zhong-Hong Zhu,*^,^ ^1^ Yan-Cheng Liu, ^1^ Hua-Hong Zou,*^, 1^ Fu-Pei Liang,*^, 1^

^1^State Key Laboratory for Chemistry and Molecular Engineering of Medicinal Resources, School of Chemistry & Pharmacy of Guangxi Normal University, Guilin 541004, P. R. China. E-mail: [gxnuchem@foxmail.com](mailto:gxnuchem@foxmail.com), 18317725515@163.com, fliangoffice@yahoo.com.

**Table of Contents:**

| **Supporting Tables** | |
| --- | --- |
| **Table S1** | Literature survey for Schiff base exchange. |
| **Table S2** | Genuine Schiff base exchange sorted by different metals. |
| **Table S3** | Crystallographic data of the complexes **1**, **2**, **3**, and **4**. |
| **Table S4** | Selected bond lengths (Å) and angles (°) of complexes **1**, **2**, **3**, and **4**. |
| **Table S5** | Hydrogen-bonds for all **Ln2** complexes. |
| **Table S6~S9** | *SHAPE* analysis of the Ln^III^ ion in **Ln2**. |
| **Table S10** | Major species assigned in the ESI-MS of **1**, **2**, **3**, and **4** in positive mode. |
| **Table S11** | Major species assigned in the ESI-MS of **1**, **2**, **3**, and **4** in negative mode. |
| **Table S12** | Time-dependent ESI-MS spectra assigned in the ESI-MS of **2**. |
| **Table S13** | Selected parameters from the fitting results of the Cole-Cole plots for **2** under 2000 Oe field. |
| **Supporting Figures** | |
| **Figure S1** | The TG curve of **Ln2** (**1**, **2**, **3**, and **4**) under heating in flowing N_2_ at 5 °C∙min^-1^ over the temperature range of 35−800 °C. |
| **Figure S2** | Powdered X-ray diffraction (XRD) patterns for complexes **1**, **2**, **3**, and **4**, respectively. |
| **Figure S3** | (a) Upper: ESI-MS spectrum of **2** crystal in negative mode (A), **L1**+Dy(NO_3_)_3_·6H_2_O was reacted in methanol at room temperature for 24 h (no addition of 2-aminomethylpyridine) (B) (positive mode), **L1**+2-aminomethylpyridine was reacted in methanol at room temperature for 24 h (no addition of Dy(NO_3_)_3_·6H_2_O) (C) (positive mode). Bottom: major species assigned in the ESI-MS. (b) The superposed simulated and observed spectra of several species for B and C (Figure 2) in negative mode. (c) The superposed simulated and observed spectra of several species for B and C (Figure S3a) in positive/negative mode. |
| **Figure S4** | (a) Positive ESI-MS spectra of **1**, **2**, **3**, and **4** in CH_3_CN (In-Source CID 0 eV). (b) Positive ESI-MS spectra of **2** in CH_3_OH (In-Source CID 0 eV). |
| **Figure S5~8** | The superposed simulated and observed spectra of several species for **1**, **2**, **3**, and **4** (In-Source CID 0 eV). |
| **Figure S9** | Negative ESI-MS spectra of **1**, **2**, **3**, and **4** in CH_3_CN (In-Source CID 0 eV). |
| **Figure S10~13** | The superposed simulated and observed spectra of several species for **1**, **2**, **3**, and **4**. |
| **Figure S14** | (a) Time-dependent ESI-MS spectra of ligand Li_2_**L1** and 2-aminomethylpyridine reaction with Dy(NO_3_)_3_·6H_2_O in methanol underroom temperature conditions. (b) Time-dependent ESI-MS spectra of ligand Li_2_**L1** and 2-aminomethylpyridine reaction with Dy(NO_3_)_3_·6H_2_O in methanol under room temperature conditions in negative mode. |
| **Figure S15** | The superposed simulated and observed spectra of time-dependent ESI-MS species for **2**. |
| **Figure S16** | (a) The **Ln2** (**1**, **2**, **3**, and **4**) complex was dissolved in an ultraviolet-visible absorption test in DMF, respectively. (b) The dinuclear compounds **2** (a), **1** (b), **3** (c), **4** (d) were dissolved in DMF solution for luminescence (excitation and emission) tests, respectively. (c) CIE chromaticity map. |
| **Figure S17** | (a) Photoluminescence in **Ln2** solid state conditions. (b) The **L2** was dissolved in DMF solution for ESI-MS measurement. (c) The L2 was dissolved in DMF solution for luminescence tests (emission). |
| **Figure S18** | The luminescence spectra of **1** dispersed in H_2_O with increasing CH_2_Cl_2_ content. |
| **Figure S19** | Temperature dependence of *χ_m_T* for all complexes. |
| **Figure S20** | *M* vs. *H* plots for **1** (a), **2** (b), **3** (c) and **4** (d). |
| **Figure S21** | The temperature dependence of the in-phase and out-of-phase ac-susceptibilities for different frequencies for **2** (a, b), **1** (c), **3** (d) and **4** (e). |
| **Figure S22** | Variable–frequency dependence of AC susceptibilities (a and b) under 2000 Oe dc field; Cole–Cole plots of AC susceptibilities for **2** (c); (d) and Arrhenius plots generated from the temperature-dependent relaxation times extracted from the ac-susceptibilities Cole–Cole fits. |
| **Figure S23** | Hysteresis loop for **2** at 2 K. |

**Table S1.** Literature survey for Schiff-base exchange.

| **Key Words** | **Number** |
| --- | --- |
| Schiff base displace | 188^a^ |
| Schiff base exchange | 17^b^ |
| Genuine Schiff base exchange | 1^c^ |

^a^Using “Schiff base displace” for topic search in “Scifinder” database from 1919 through Dec. 2, 2018. 188 papers were found; ^b^ Using “Schiff base exchange” for topic search in “Scifinder” database from 1965 through Dec. 2, 2018. 17 papers were found; ^b^ Furthermore, 1 of the contain genuine Schiff base exchange.

**Table S2.** Genuine Schiff-base exchange sorted by different metals.

| **No** | **Reaction of ligand and metal salt** | **Mechanism study** | ***Ref.*** |
| --- | --- | --- | --- |
| 1 | 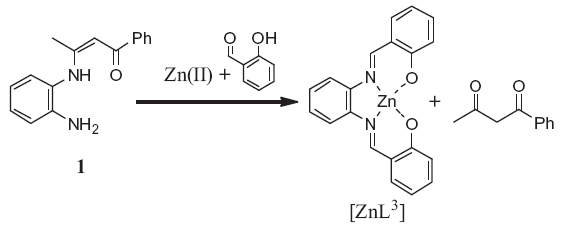 | No | 1 |
| 2 | **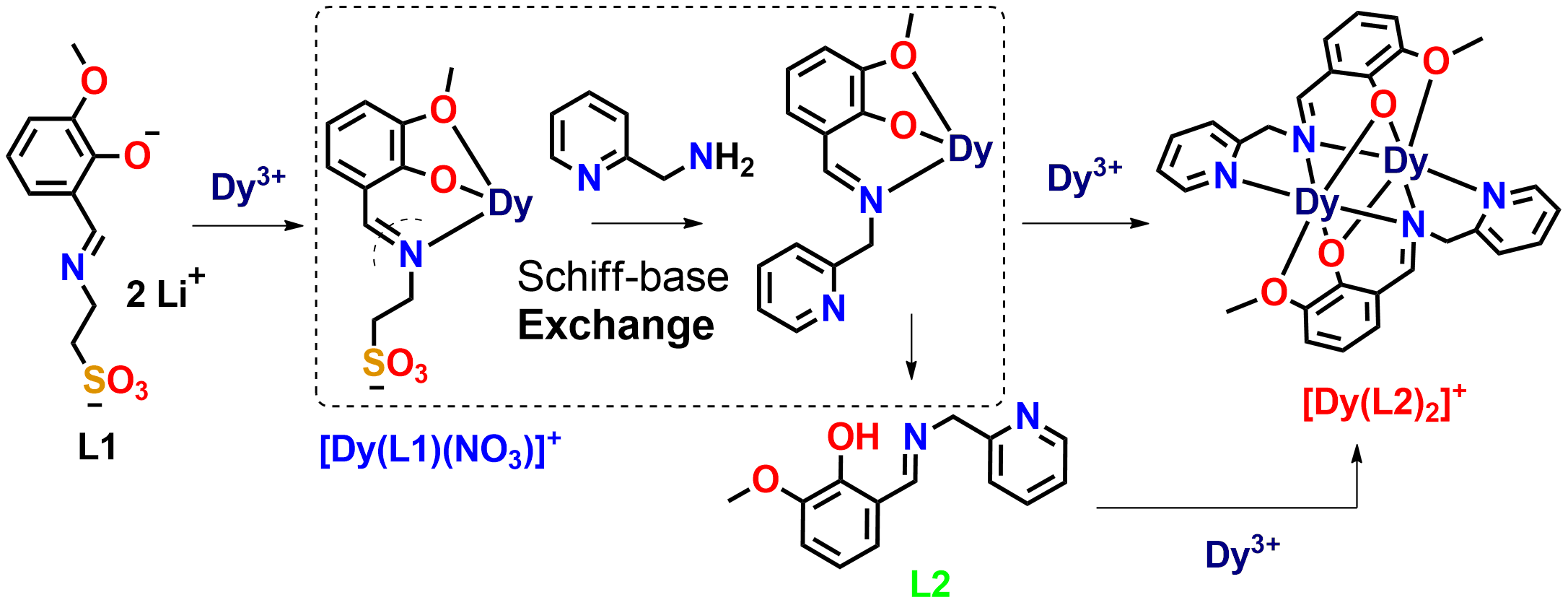** | Yes | ***This work*** |

1. Q. Meng, J. K. Clegg, A, J. Brock, K. A. Jolliffe, L. F. Lindoy, G. W, *Polyhedron*, **2014**, *74*, 113–121.
2. ***This work***.

**Table S3.** Crystallographic data of the complexes **1**, **2**, **3**, and **4**.

| **Complex** | **1** | **2** | **3** | **4** |
| --- | --- | --- | --- | --- |
| Formula | C_30_H_28_Tb_2_N_8_O_18_ | C_30_H_28_Dy_2_N_8_O_18_ | C_30_H_28_Ho_2_N_8_O_18_ | C_30_H_28_Er_2_N_8_O_18_ |
| Formula weight | 1106.44 | 1113.60 | 1118.46 | 1123.12 |
| *T* (K) | 293(2) | 293(2) | 293(2) | 293(2) |
| Crystal system | Triclinic | Triclinic | Triclinic | Triclinic |
| Space group | *P*-1 | *P*-1 | *P*-1 | *P*-1 |
| *a* (Å) | 9.9763(2) | 9.9718(4) | 9.9472(4) | 9.9436(5) |
| *b* (Å) | 10.0494(2) | 10.0164(3) | 9.9963(3) | 9.9985(9) |
| *c* (Å) | 11.1816(3) | 11.1731(5) | 11.1721(5) | 11.1634(8) |
| *α* (°) | 67.201(2) | 67.350(4) | 67.388(4) | 67.488(8) |
| *β* (°) | 72.780(2) | 72.714(4) | 72.530(3) | 72.552(6) |
| *γ* (°) | 87.564(2) | 87.627(3) | 87.401(3) | 87.384(6) |
| *V* (Å^3^) | 983.79(4) | 980.02(7) | 975.10(7) | 975.12(12) |
| *Z* | 1 | 1 | 1 | 1 |
| *D_c_*(g cm^–3^) | 1.868 | 1.887 | 1.905 | 1.913 |
| *μ* (mm^–1^) | 3.650 | 3.868 | 4.113 | 4.359 |
| Reflns coll. | 14988 | 12214 | 13154 | 11622 |
| Unique reflns | 3652 | 3642 | 3623 | 3624 |
| *R*_int_ | 0.0328 | 0.0321 | 0.0372 | 0.0606 |
| *^a^R*_1_[*I* ≥ 2*σ*(*I*)] | 0.0220 | 0.0228 | 0.0230 | 0.0285 |
| *^b^wR*_2_(all data) | 0.0587 | 0.0603 | 0.0603 | 0.0758 |
| GOF | 1.105 | 1.094 | 1.075 | 1.089 |

^a^*R*_1_ = Σ||*F*_o_|-|*F*_c_||/Σ|F_o_|, ^b^w*R*_2_ = [Σw(*F*_o_^2^-*F*_c_^2^)^2^/Σw(*F*_o_^2^)^2^]^1/2^

**Table S4.** Selected bond lengths (Å) and angles (°) of complexes **1**, **2**, **3**, and **4**.

| **1** | | | | | | |
| --- | --- | --- | --- | --- | --- | --- |
| **Bond lengths (Å)** | | | | | | |
| Tb1—O2^i^ | 2.380(2) | Tb1—O1^i^ | 2.438(2) | Tb1—O4 | 2.501(3) | |
| Tb1—O2 | 2.308(2) | Tb1—O6 | 2.454(3) | Tb1—N1 | 2.439(3) | |
| Tb1—O7 | 2.480(3) | Tb1—O3 | 2.490(3) | Tb1—N2 | 2.522(3) | |
| **Bond angles (°)** | | | | | | |
| O2—Tb1—O2^i^ | 74.16(9) | O2—Tb1—N1 | 77.56(9) | O6—Tb1—O7 | | 51.59(9) |
| O2^i^—Tb1—O7 | 105.00(9) | O2—Tb1—N2 | 137.29(9) | O6—Tb1—O3 | | 153.47(10) |
| O2—Tb1—O7 | 74.27(9) | O2^i^—Tb1—N2 | 143.02(10) | O6—Tb1—O4 | | 136.46(10) |
| O2—Tb1—O1^i^ | 139.65(8) | O7—Tb1—O3 | 145.19(11) | O6—Tb1—N2 | | 75.97(10) |
| O2^i^—Tb1—O1^i^ | 66.27(8) | O7—Tb1—O4 | 141.54(10) | O3—Tb1—O4 | | 50.65(10) |
| O2—Tb1—O6 | 106.07(9) | O7—Tb1—N2 | 74.98(10) | O4—Tb1—N2 | | 73.36(10) |
| O2^i^—Tb1—O6 | 75.99(9) | O1^i^—Tb1—O7 | 122.80(9) | N1—Tb1—O7 | | 77.20(10) |
| O2—Tb1—O3 | 73.57(10) | O1^i^—Tb1—O6 | 72.27(9) | N1—Tb1—O6 | | 123.05(10) |
| O2^i^—Tb1—O3 | 78.55(9) | O1^i^—Tb1—O3 | 90.76(11) | N1—Tb1—O3 | | 83.19(11) |
| O2—Tb1—O4 | 117.44(9) | O1^i^—Tb1—O4 | 73.72(9) | N1—Tb1—O4 | | 70.79(10) |
| O2^i^—Tb1—O4 | 113.41(9) | O1^i^—Tb1—N1 | 138.44(10) | N1—Tb1—N2 | | 67.24(11) |
| O2^i^—Tb1—N1 | 149.67(10) | Symmetry code: (i) -*x*+1, -*y*+1, -*z*+1. | | | | |
| **2** | | | | | | |
| **Bond lengths (Å)** | | | | | | |
| Dy1—O2^i^ | 2.364(2) | Dy1—O1^i^ | 2.423(3) | Dy1—O6 | | 2.488(3) |
| Dy1—O2 | 2.298(2) | Dy1—O4 | 2.443(3) | Dy1—N1 | | 2.426(3) |
| Dy1—O3 | 2.470(3) | Dy1—O7 | 2.475(3) | Dy1—N2 | | 2.516(3) |
| **Bond angles (°)** | | | | | | |
| O2—Dy1—O2^i^ | 73.74(10) | O2—Dy1—N1 | 77.89(10) | O4—Dy1—O3 | | 51.94(10) |
| O2^i^—Dy1—O3 | 105.25(10) | O2—Dy1—N2 | 137.67(10) | O4—Dy1—O7 | | 153.14(11) |
| O2—Dy1—O3 | 74.21(10) | O2^i^—Dy1—N2 | 142.86(10) | O4—Dy1—O6 | | 136.53(10) |
| O2—Dy1—O1^i^ | 139.55(9) | O3—Dy1—O7 | 144.96(11) | O4—Dy1—N2 | | 75.96(10) |
| O2^i^—Dy1—O1^i^ | 66.58(9) | O3—Dy1—O6 | 141.13(11) | O7—Dy1—O6 | | 50.96(10) |
| O2—Dy1—O4 | 105.67(9) | O3—Dy1—N2 | 74.80(11) | O7—Dy1—N2 | | 123.32(10) |
| O2^i^—Dy1—O4 | 75.80(9) | O1^i^—Dy1—O3 | 123.11(10) | O6—Dy1—N2 | | 73.37(10) |
| O2—Dy1—O7 | 73.55(10) | O1^i^—Dy1—O4 | 72.37(10) | N1—Dy1—O3 | | 76.62(11) |
| O2^i^—Dy1—O7 | 78.35(10) | O1^i^—Dy1—O7 | 90.74(11) | N1—Dy1—O4 | | 122.96(10) |
| O2—Dy1—O6 | 117.76(9) | O1^i^—Dy1—O6 | 73.56(10) | N1—Dy1—O7 | | 83.57(11) |
| O2^i^—Dy1—O6 | 113.58(10) | O1^i^—Dy1—N1 | 138.31(10) | N1—Dy1—O6 | | 70.93(11) |
| O2^i^—Dy1—N1 | 149.67(10) | O1^i^—Dy1—N2 | 82.15(10) | N1—Dy1—N2 | | 67.39(11) |
| Dy1—O2—Dy1^i^ | 106.26(10) | Symmetry code: (i) -*x*+1, -*y*+1, -*z*+1. | | | | |
| **3** | | | | | | |
| **Bond lengths (Å)** | | | | | | |
| Ho1—O2 | 2.292(2) | Ho1—O7 | 2.431(3) | Ho1—O3 | | 2.455(3) |
| Ho1—O2^i^ | 2.351(2) | Ho1—O6 | 2.458(3) | Ho1—N2 | | 2.501(3) |
| Ho1—O1^i^ | 2.414(2) | Ho1—O4 | 2.478(3) | Ho1—N1 | | 2.411(3) |
| **Bond angles (°)** | | | | | | |
| O2—Ho1—O2^i^ | 73.52(9) | O2^i^—Ho1—N2 | 142.33(10) | O6—Ho1—O4 | | 141.21(11) |
| O2^i^—Ho1—O1^i^ | 66.88(8) | O2—Ho1—N1 | 78.18(10) | O6—Ho1—N2 | | 75.07(11) |
| O2—Ho1—O1^i^ | 139.60(9) | O2^i^—Ho1—N1 | 149.75(10) | O4—Ho1—N2 | | 73.25(11) |
| O2^i^—Ho1—O7 | 75.54(9) | O1^i^—Ho1—O7 | 72.38(9) | O3—Ho1—O6 | | 145.11(11) |
| O2—Ho1—O7 | 105.63(9) | O1^i^—Ho1—O6 | 123.15(10) | O3—Ho1—O4 | | 51.07(10) |
| O2—Ho1—O6 | 74.22(10) | O1^i^—Ho1—O4 | 73.22(10) | O3—Ho1—N2 | | 123.41(11) |
| O2^i^—Ho1—O6 | 104.97(10) | O1^i^—Ho1—O3 | 90.55(11) | N1—Ho1—O1^i^ | | 137.91(10) |
| O2—Ho1—O4 | 118.00(9) | O1^i^—Ho1—N2 | 81.48(10) | N1—Ho1—O7 | | 123.20(10) |
| O2^i^—Ho1—O4 | 113.77(10) | O7—Ho1—O6 | 52.01(9) | N1—Ho1—O6 | | 76.86(11) |
| O2^i^—Ho1—O3 | 78.43(10) | O7—Ho1—O4 | 136.32(10) | N1—Ho1—O4 | | 70.84(11) |
| O2—Ho1—O3 | 73.65(10) | O7—Ho1—O3 | 152.89(11) | N1—Ho1—O3 | | 83.61(11) |
| O2—Ho1—N2 | 138.28(10) | O7—Ho1—N2 | 75.77(10) | N1—Ho1—N2 | | 67.82(11) |
| Ho1—O2—Ho1^i^ | 106.48(9) | Symmetry code: (i) -*x*+1, -*y*, -*z*+1. | | | | |
| **4** | | | | | | |
| **Bond lengths (Å)** | | | | | | |
| Er1—O7 | 2.475(3) | Er1—O2 | 2.288(3) | Er1—N1 | | 2.401(4) |
| Er1—N2 | 2.494(4) | Er1—O2^i^ | 2.348(3) | Er1—O6 | | 2.453(3) |
| Er1—O4 | 2.427(3) | Er1—O3 | 2.452(3) | Er1—O1^i^ | | 2.408(3) |
| **Bond angles (°)** | | | | | | |
| O7—Er1—N2 | 73.47(12) | O2^i^—Er1—O3 | 105.24(12) | N1—Er1—N2 | | 68.13(13) |
| O4—Er1—O7 | 136.30(12) | O2—Er1—O3 | 74.15(11) | N1—Er1—O4 | | 123.25(12) |
| O4—Er1—N2 | 75.45(13) | O2^i^—Er1—N1 | 149.89(12) | N1—Er1—O3 | | 76.53(13) |
| O4—Er1—O3 | 52.38(12) | O2—Er1—N1 | 78.26(12) | N1—Er1—O6 | | 83.67(14) |
| O4—Er1—O6 | 152.78(14) | O2^i^—Er1—O6 | 78.43(12) | N1—Er1—O1^i^ | | 137.68(12) |
| O2^i^—Er1—O7 | 113.78(11) | O2—Er1—O6 | 73.61(12) | O6—Er1—O7 | | 51.13(12) |
| O2—Er1—O7 | 118.02(11) | O2—Er1—O1^i^ | 139.78(11) | O6—Er1—N2 | | 123.74(12) |
| O2—Er1—N2 | 138.49(12) | O2^i^—Er1—O1^i^ | 67.01(11) | O1^i^—Er1—O7 | | 73.12(12) |
| O2^i^—Er1—N2 | 141.88(12) | O3—Er1—O7 | 140.94(13) | O1^i^—Er1—N2 | | 81.05(12) |
| O2^i^—Er1—O4 | 75.42(11) | O3—Er1—N2 | 74.89(13) | O1^i^—Er1—O4 | | 72.34(11) |
| O2—Er1—O4 | 105.62(11) | O3—Er1—O6 | 144.89(13) | O1^i^—Er1—O3 | | 123.38(12) |
| O2—Er1—O2^i^ | 73.58(12) | N1—Er1—O7 | 70.82(13) | O1^i^—Er1—O6 | | 90.63(13) |
| Er1—O2—Er1^i^ | 106.42(12) | Symmetry code: (i) -*x*+2, -*y*+1, -*z*+1. | | | | |

**Table S5.** Hydrogen-bonds for all **Ln2** complexes.

| **1** | | |
| --- | --- | --- |
| **Hydrogen bond** | **Distance^a^, Å** | **Angle^b^, °** |
| C_1_−H_1C_∙∙∙O_5_ | 2.457 | 164 |
| C_11_−H_11_∙∙∙O_8_ | 2.557 | 153 |
| **2** | | |
| **Hydrogen bond** | **Distance^a^, Å** | **Angle^b^, °** |
| C_1_−H_1C_∙∙∙O_5_ | 2.461 | 163 |
| C_11_−H_11_∙∙∙O_8_ | 2.558 | 154 |
| **3** | | |
| **Hydrogen bond** | **Distance^a^, Å** | **Angle^b^, °** |
| C_1_−H_1C_∙∙∙O_5_ | 2.469 | 163 |
| C_11_−H_11_∙∙∙O_8_ | 2.549 | 155 |
| **4** | | |
| **Hydrogen bond** | **Distance^a^, Å** | **Angle^b^, °** |
| C_1_−H_1C_∙∙∙O_5_ | 2.487 | 163 |
| C_11_−H_11_∙∙∙O_8_ | 2.561 | 155 |

^a^ Distance between acceptor and donor; ^b^ Angle of acceptor−hydrogen−donor.

**Table S6.** *SHAPE* analysis of the Tb^III^ ion in **1**.

| **Label** | **Shape** | **Symmetry** | **Distortion(**^o^**)** |
| --- | --- | --- | --- |
| EP-9 | *D*_9h_ | Enneagon | 35.156 |
| OPY-9 | *C*_8v_ | Octagonal pyramid | 21.169 |
| HBPY-9 | *D*_7h_ | Heptagonal bipyramid | 18.084 |
| JTC-9 | *C*_3v_ | Johnson triangular cupola J3 | 16.519 |
| JCCU-9 | *C*_4v_ | Capped cube J8 | 10.696 |
| CCU-9 | *C*_4v_ | Spherical-relaxed capped cube | 8.471 |
| JCSAPR-9 | *C*_4v_ | Capped square antiprism J10 | 2.921 |
| CSAPR-9 | *C*_4v_ | Spherical capped square antiprism | 1.905 |
| JTCTPR-9 | *D*_3h_ | Tricapped trigonal prism J51 | 4.703 |
| TCTPR-9 | *D*_3h_ | Spherical tricapped trigonal prism | 2.923 |
| JTDIC-9 | *C*_3v_ | Tridiminished icosahedron J63 | 13.136 |
| HH-9 | *C*_2v_ | Hula-hoop | 8.691 |
| MFF-9 | *C*_s_ | Muffin | 1.526 |

**Table S7.** *SHAPE* analysis of the Dy^III^ ion in **2**.

| **Label** | **Shape** | **Symmetry** | **Distortion(**^o^**)** |
| --- | --- | --- | --- |
| EP-9 | *D*_9h_ | Enneagon | 35.228 |
| OPY-9 | *C*_8v_ | Octagonal pyramid | 21.263 |
| HBPY-9 | *D*_7h_ | Heptagonal bipyramid | 18.116 |
| JTC-9 | *C*_3v_ | Johnson triangular cupola J3 | 16.402 |
| JCCU-9 | *C*_4v_ | Capped cube J8 | 10.789 |
| CCU-9 | *C*_4v_ | Spherical-relaxed capped cube | 8.587 |
| JCSAPR-9 | *C*_4v_ | Capped square antiprism J10 | 2.865 |
| CSAPR-9 | *C*_4v_ | Spherical capped square antiprism | 1.845 |
| JTCTPR-9 | *D*_3h_ | Tricapped trigonal prism J51 | 4.608 |
| TCTPR-9 | *D*_3h_ | Spherical tricapped trigonal prism | 2.890 |
| JTDIC-9 | *C*_3v_ | Tridiminished icosahedron J63 | 13.130 |
| HH-9 | *C*_2v_ | Hula-hoop | 8.767 |
| MFF-9 | *C*_s_ | Muffin | 1.466 |

**Table S8.** *SHAPE* analysis of the Ho^III^ ion in **3**.

| **Label** | **Shape** | **Symmetry** | **Distortion(**^o^**)** |
| --- | --- | --- | --- |
| EP-9 | *D*_9h_ | Enneagon | 35.401 |
| OPY-9 | *C*_8v_ | Octagonal pyramid | 21.371 |
| HBPY-9 | *D*_7h_ | Heptagonal bipyramid | 18.148 |
| JTC-9 | *C*_3v_ | Johnson triangular cupola J3 | 16.358 |
| JCCU-9 | *C*_4v_ | Capped cube J8 | 10.816 |
| CCU-9 | *C*_4v_ | Spherical-relaxed capped cube | 8.634 |
| JCSAPR-9 | *C*_4v_ | Capped square antiprism J10 | 2.801 |
| CSAPR-9 | *C*_4v_ | Spherical capped square antiprism | 1.799 |
| JTCTPR-9 | *D*_3h_ | Tricapped trigonal prism J51 | 4.568 |
| TCTPR-9 | *D*_3h_ | Spherical tricapped trigonal prism | 2.866 |
| JTDIC-9 | *C*_3v_ | Tridiminished icosahedron J63 | 13.119 |
| HH-9 | *C*_2v_ | Hula-hoop | 8.773 |
| MFF-9 | *C*_s_ | Muffin | 1.427 |

**Table S9.** *SHAPE* analysis of the Er^III^ ion in **4**.

| **Label** | **Shape** | **Symmetry** | **Distortion(**^o^**)** |
| --- | --- | --- | --- |
| EP-9 | *D*_9h_ | Enneagon | 35.466 |
| OPY-9 | *C*_8v_ | Octagonal pyramid | 21.456 |
| HBPY-9 | *D*_7h_ | Heptagonal bipyramid | 18.116 |
| JTC-9 | *C*_3v_ | Johnson triangular cupola J3 | 16.327 |
| JCCU-9 | *C*_4v_ | Capped cube J8 | 10.903 |
| CCU-9 | *C*_4v_ | Spherical-relaxed capped cube | 8.728 |
| JCSAPR-9 | *C*_4v_ | Capped square antiprism J10 | 2.758 |
| CSAPR-9 | *C*_4v_ | Spherical capped square antiprism | 1.760 |
| JTCTPR-9 | *D*_3h_ | Tricapped trigonal prism J51 | 4.512 |
| TCTPR-9 | *D*_3h_ | Spherical tricapped trigonal prism | 2.852 |
| JTDIC-9 | *C*_3v_ | Tridiminished icosahedron J63 | 13.089 |
| HH-9 | *C*_2v_ | Hula-hoop | 8.792 |
| MFF-9 | *C*_s_ | Muffin | 1.386 |

**Thermal analysis**

The thermal stability of the complexes **1**, **2**, **3**, and **4** at a heating rate of 5 °C/min in a flowing N_2_ at 35-800 °C was investigated. The weight loss behaviours of thesecomplexes are very similar and exhibit several successive weight loss steps due to their structural similarities. The first weight loss of 16.21%, 16.17%, 16.09%, and 16.01% for complexes **1**, **2**, **3**, and **4**, respectively, maybe corresponds to the release of two free methanol and two coordinated nitrate anion (*calc*. 16.99% for **1**, 16.88% for **2**, 16.81% for **3,** and 16.73% for **4**) (Figure S1).And then the decomposition of the organic ligands with weight loss of 43.49%, 43.21%, 42.97%, and 42.85% for complexes **1**, **2**, **3**, and **4** (*calc*. 43.61% for **1**, 43.33% for **2**, 43.14% for **3,** and 42.96% for **4**), respectively.The final decomposition products may be the corresponding metal oxide. The thermal behaviors is in agreement with the formulae of these complexes.The PXRD patterns of **1**, **2**, **3**, and **4** match the simulated ones using the single crystal structure data (Figures S2). These results confirm the single-phase nature of the bulk.


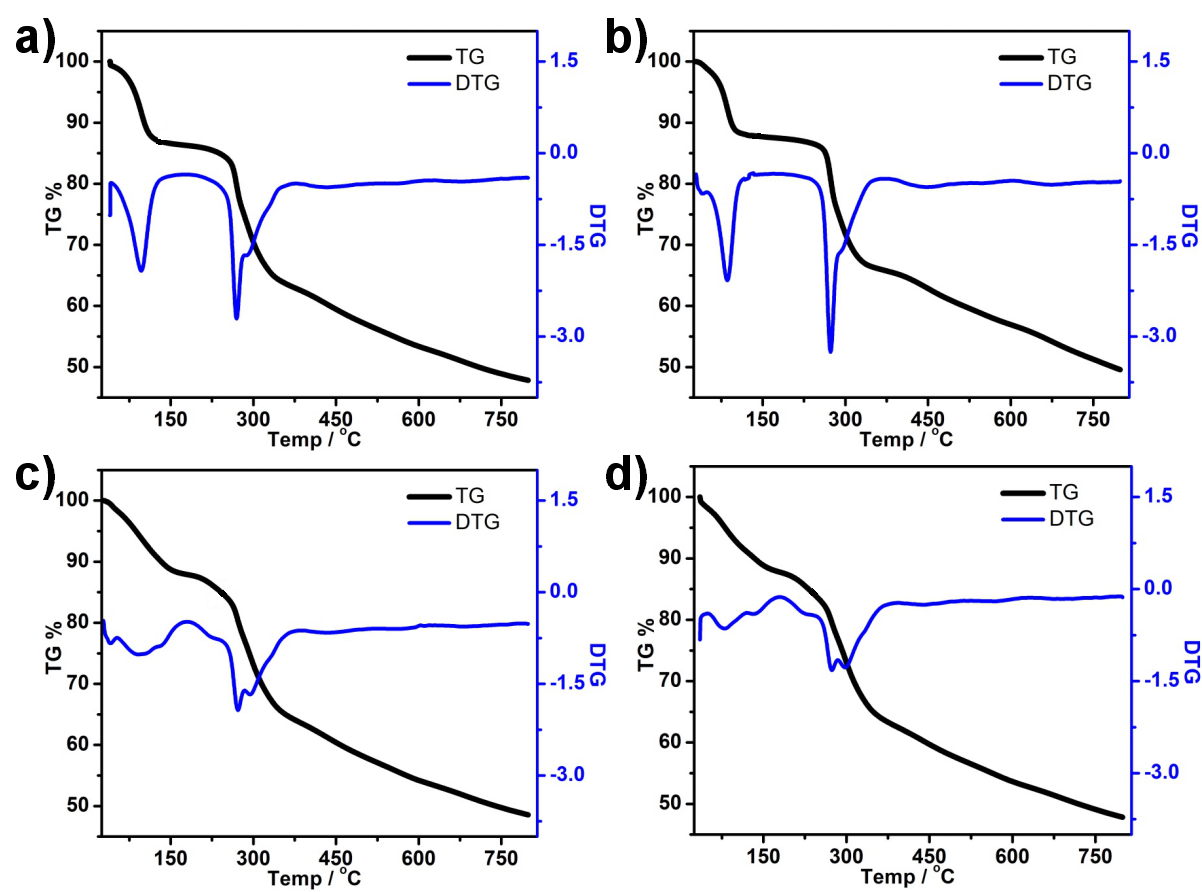


**Figure S1.** The TG curve of **Ln2** (**1**, **2**, **3**, and **4**) under heating in flowing N_2_ at 5 °C∙min^-1^ over the temperature range of 35-800 °C.


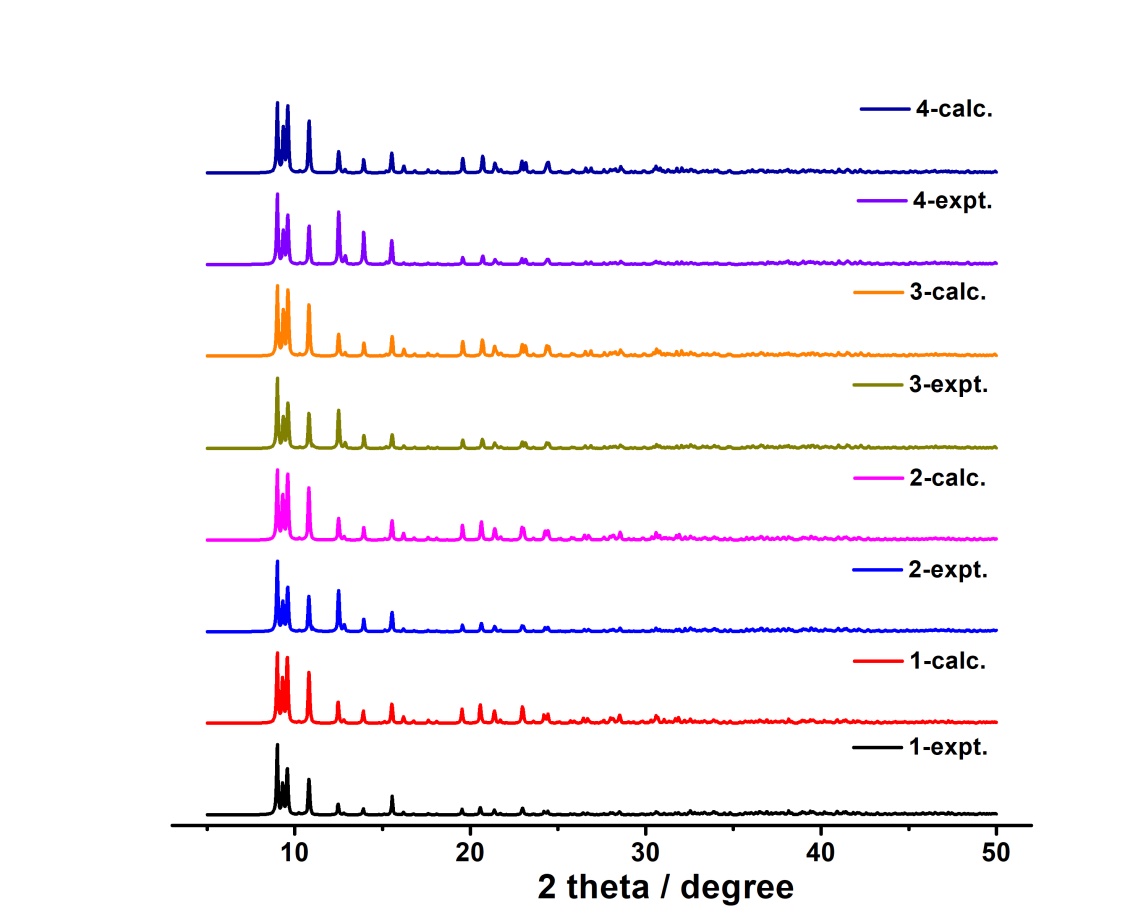


**Figure S2.** Powdered X-ray diffraction (XRD) patterns for complexes **1**, **2**, **3**, and **4**, respectively.


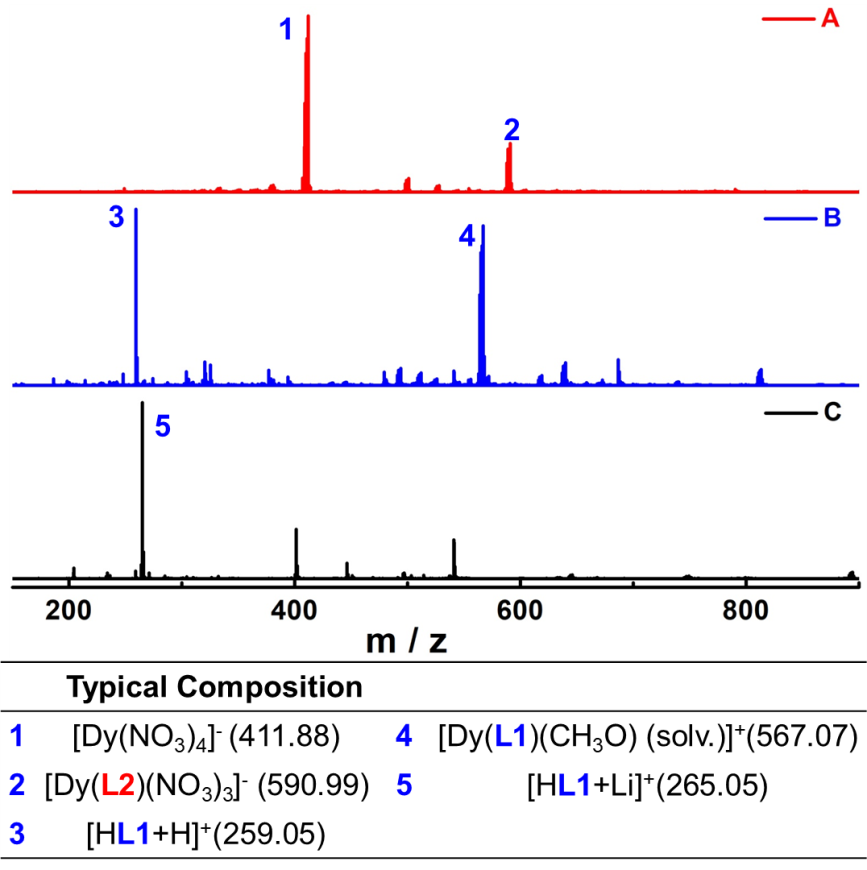


**Figure S3a.** Upper: ESI-MS spectrum of **2** crystal in negative mode (A), **L1**+Dy(NO_3_)_3_·6H_2_O was reacted in methanol at room temperature for 24 h (no addition of 2-aminomethylpyridine) (B) (positive mode), **L1**+2-aminomethylpyridine was reacted in methanol at room temperature for 24 h (no addition of Dy(NO_3_)_3_·6H_2_O) (C) (positive mode). Bottom: major species assigned in the ESI-MS.


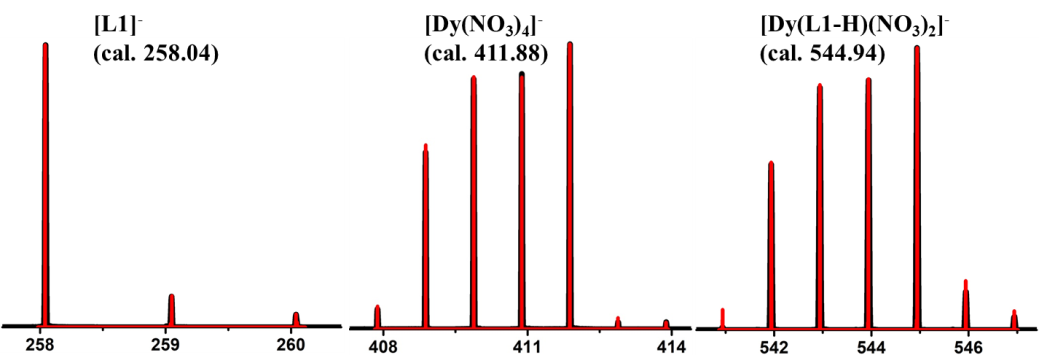


**Figure S3b.** The superposed simulated and observed spectra of several species for B and C (Figure 2) in negative mode.


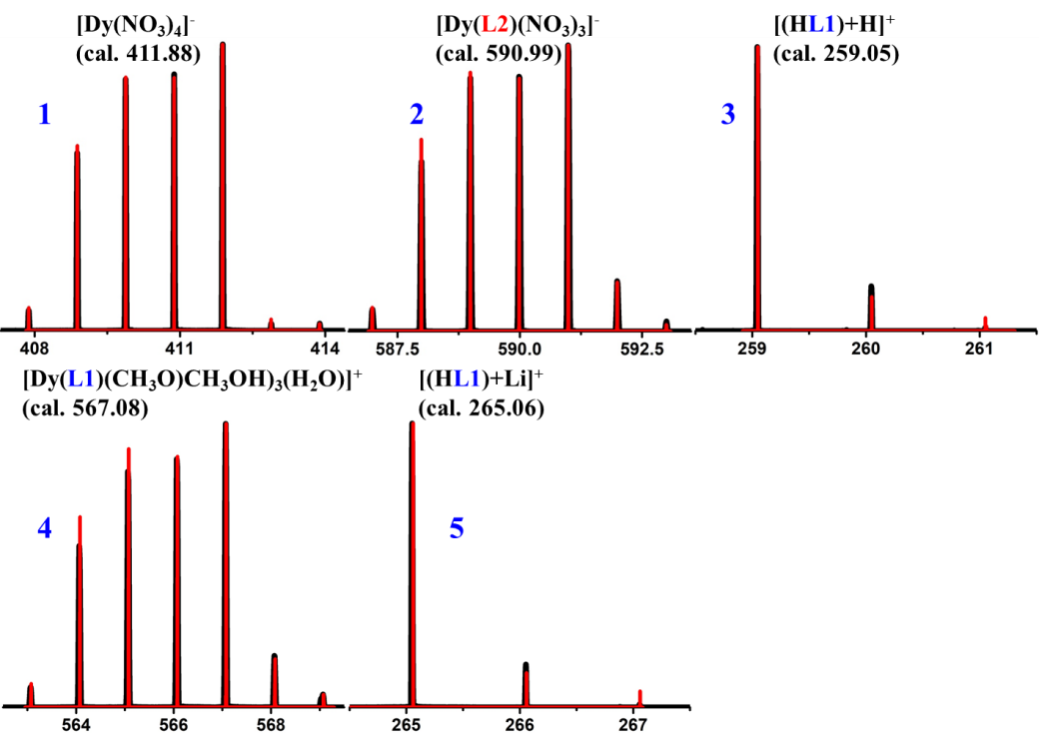


**Figure S3c.** The superposed simulated and observed spectra of several species for B and C (Figure S3a) in positive/negative mode.


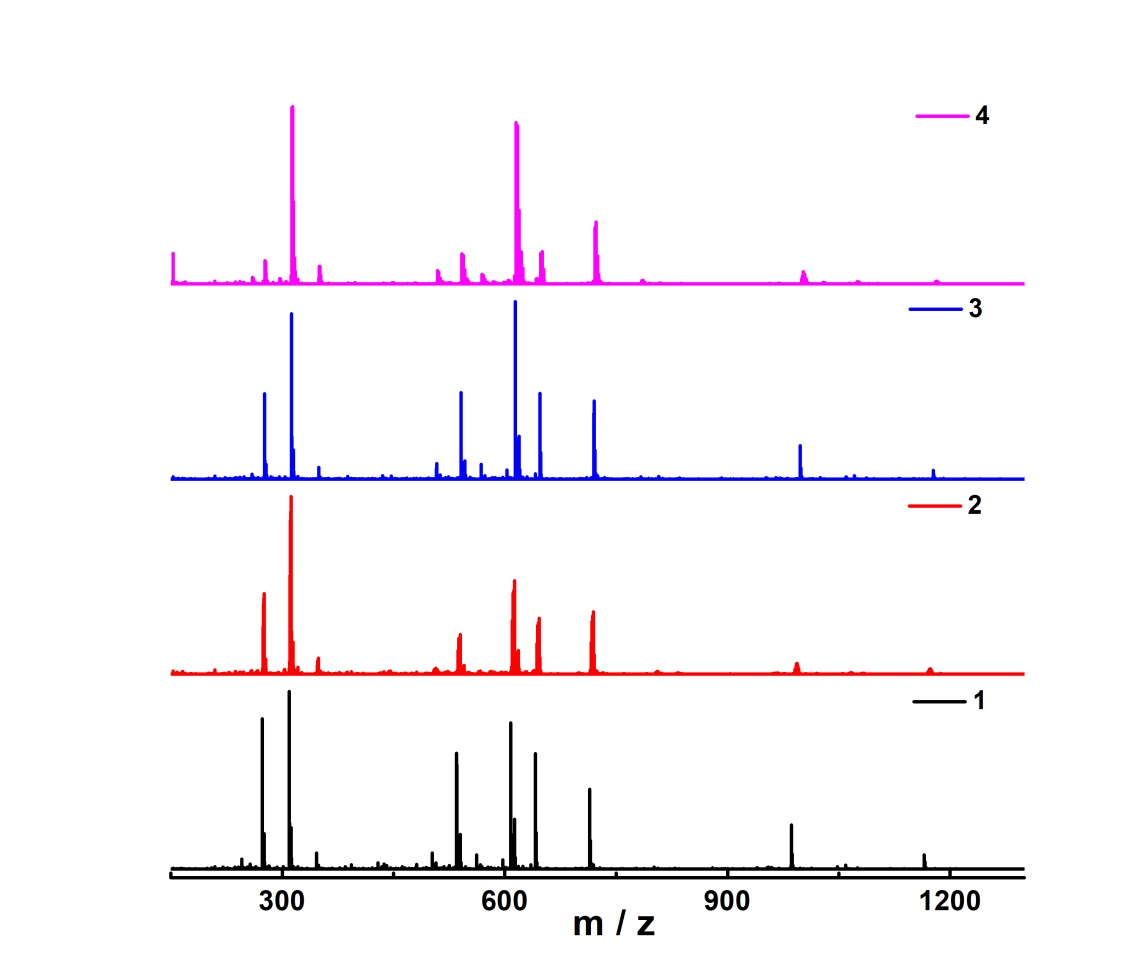


**Figure S4a.** Positive ESI-MS spectra of **Tb2**, **Dy2**, **Ho2** and **Er2** in DMF (In-Source CID 0 eV).


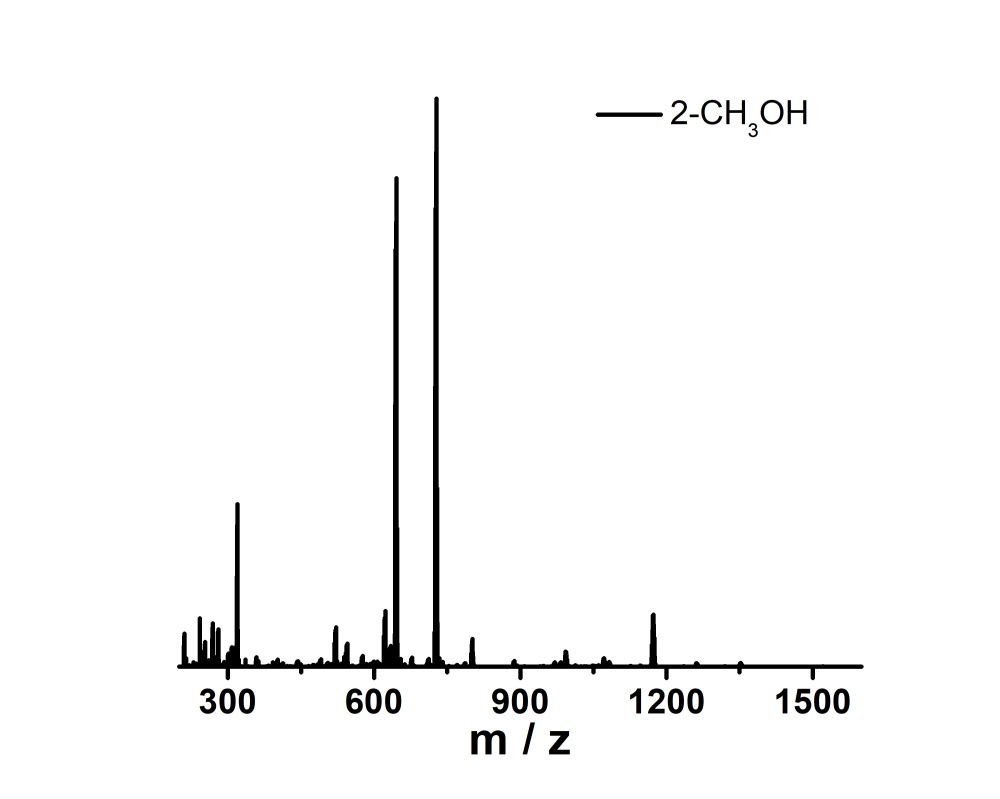


**Figure S4b.** Positive ESI-MS spectra of **2** in CH_3_OH (In-Source CID 0 eV).

**Table S10**. Major species assigned in the ESI-MS of **1**, **2**, **3**, and **4** in positive mode.

| **1** | | | |
| --- | --- | --- | --- |
| Peaks | Relative Intensity | Obs. *m/z* | Calc. *m/z* |
| [Tb(L2)(DMF)_2_]^2+^ | 0.847 | 273.06 | 273.07 |
| [Tb(L2)(DMF)_3_]^2+^ | 1 | 309.59 | 309.59 |
| [Tb(L2)(DMF)_4_]^2+^ | 0.089 | 346.11 | 346.12 |
| [Tb(L2)(NO_3_)(DMF)]^+^ | 0.652 | 535.06 | 535.06 |
| [Tb(L2)(NO_3_)(DMF)_2_]^+^ | 0.823 | 608.12 | 608.12 |
| [Tb(L2)_2_]^+^ | 0.649 | 641.11 | 641.12 |
| [Tb(L2)_2_(DMF)]^+^ | 0.449 | 714.16 | 714.17 |
| [Tb_2_(L2)_2_(NO_3_)_3_]^+^ | 0.249 | 986.01 | 986.01 |
| [Tb_2_(L2)_3_(NO_3_)_2_]^+^ | 0.079 | 1165.13 | 1165.12 |
| **2** | | | |
| [Dy(L2)(DMF)_2_]^2+^ | 0.454 | 275.56 | 275.57 |
| [Dy(L2)(DMF)_3_]^2+^ | 1 | 312.09 | 312.10 |
| [Dy(L2)(DMF)_4_]^2+^ | 0.090 | 348.62 | 348.62 |
| [Dy(L2)(NO_3_)(DMF)]^+^ | 0.221 | 540.06 | 540.07 |
| [Dy(L2)(NO_3_)(DMF)_2_]^+^ | 0.526 | 613.12 | 613.12 |
| [Dy(L2)_2_]^+^ | 0.315 | 646.12 | 646.12 |
| [Dy(L2)_2_(DMF)]^+^ | 0.350 | 719.17 | 719.18 |
| [Dy_2_(L2)_2_(NO_3_)_3_]^+^ | 0.058 | 993.00 | 993.01 |
| [Dy_2_(L2)_3_(NO_3_)_2_]^+^ | 0.029 | 1173.12 | 1173.12 |
| **3** | | | |
| [Ho(L2)(DMF)_2_]^2+^ | 0.481 | 276.07 | 276.07 |
| [Ho(L2)(DMF)_3_]^2+^ | 0.932 | 312.59 | 312.60 |
| [Ho(L2)(DMF)_4_]^2+^ | 0.065 | 349.12 | 349.12 |
| [Ho(L2)(NO_3_)(DMF)]^+^ | 0.488 | 541.07 | 541.07 |
| [Ho(L2)(NO_3_)(DMF)_2_]^+^ | 1 | 614.12 | 614.12 |
| [Ho(L2)_2_]^+^ | 0.483 | 647.12 | 647.13 |
| [Ho(L2)_2_(DMF)]^+^ | 0.439 | 720.17 | 720.18 |
| [Ho_2_(L2)_2_(NO_3_)_3_]^+^ | 0.187 | 998.02 | 998.02 |
| [Ho_2_(L2)_3_(NO_3_)_2_]^+^ | 0.048 | 1177.12 | 1177.13 |
| **4** | | | |
| [Er(L2)(DMF)_2_]^2+^ | 0.131 | 276.56 | 276.57 |
| [Er(L2)(DMF)_3_]^2+^ | 1 | 314.09 | 314.10 |
| [Er(L2)(DMF)_4_]^2+^ | 0.100 | 350.62 | 350.62 |
| [Er(L2)(NO_3_)(DMF)]^+^ | 0.170 | 542.06 | 542.07 |
| [Er(L2)(NO_3_)(DMF)_2_]^+^ | 0.909 | 615.12 | 615.12 |
| [Er(L2)_2_]^+^ | 0.183 | 650.13 | 650.13 |
| [Er(L2)_2_(DMF)]^+^ | 0.350 | 723.17 | 723.18 |
| [Er_2_(L2)_2_(NO_3_)_3_]^+^ | 0.069 | 1002.03 | 1002.02 |
| [Er_2_(L2)_3_(NO_3_)_2_]^+^ | 0.016 | 1181.14 | 1181.13 |


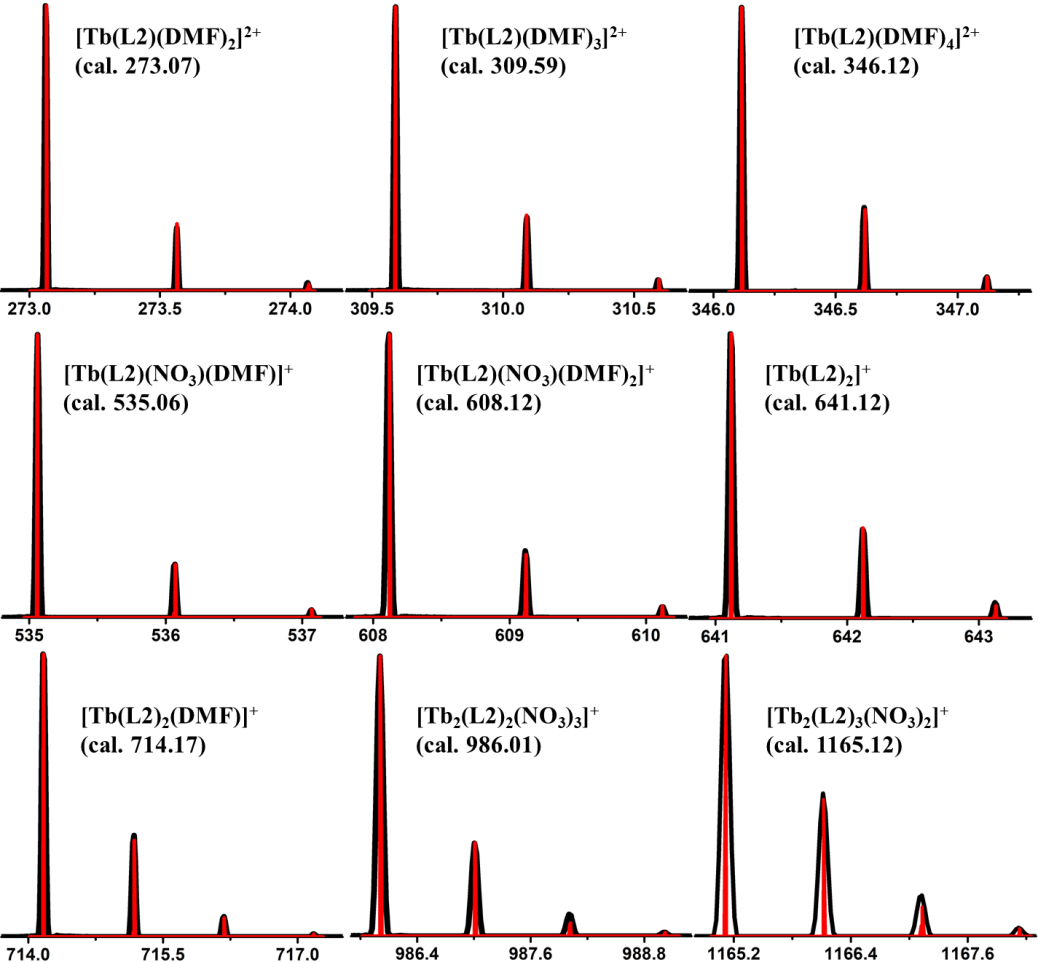


**Figure S5.** The superposed simulated and observed spectra of several species for **1** (In-Source CID 0 eV).


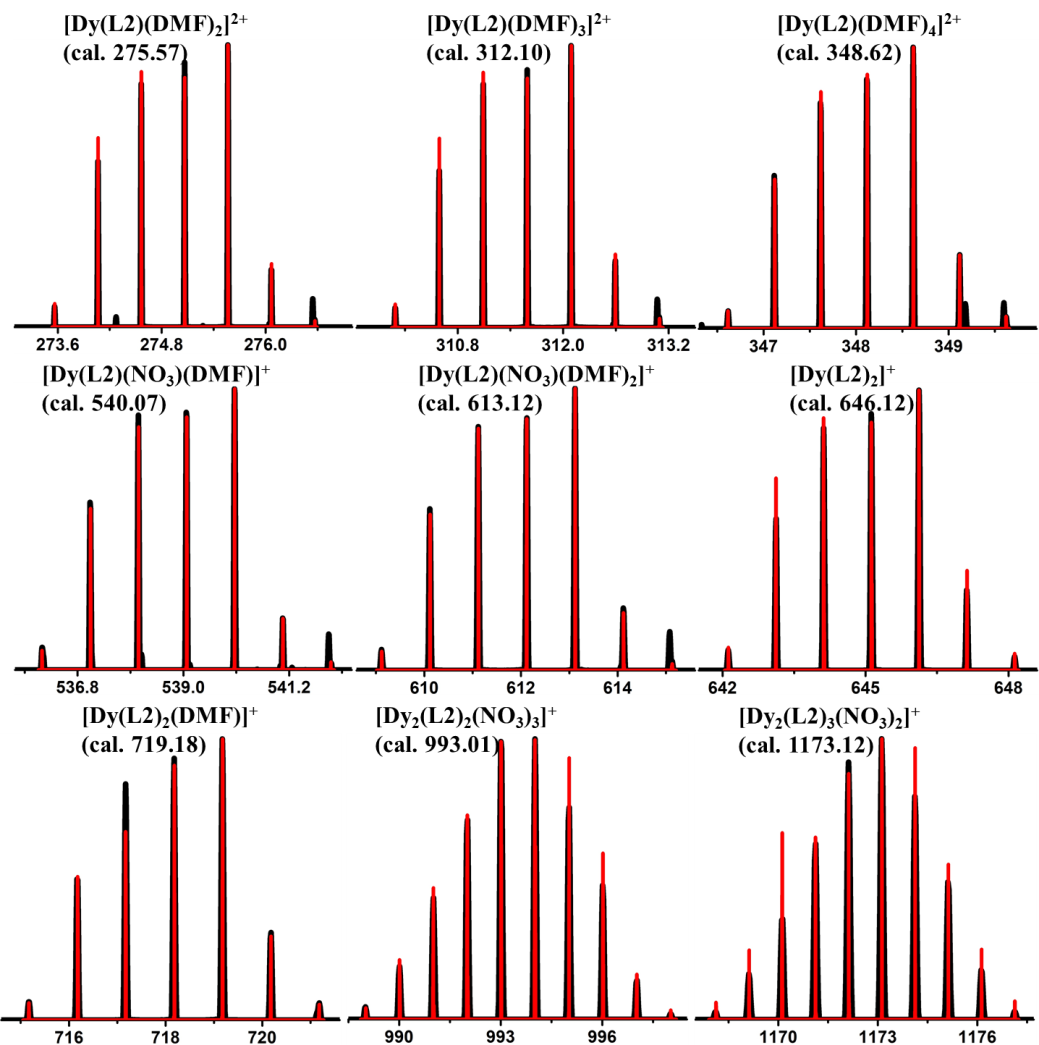


**Figure S6.** The superposed simulated and observed spectra of several species for **2** (In-Source CID 0 eV).


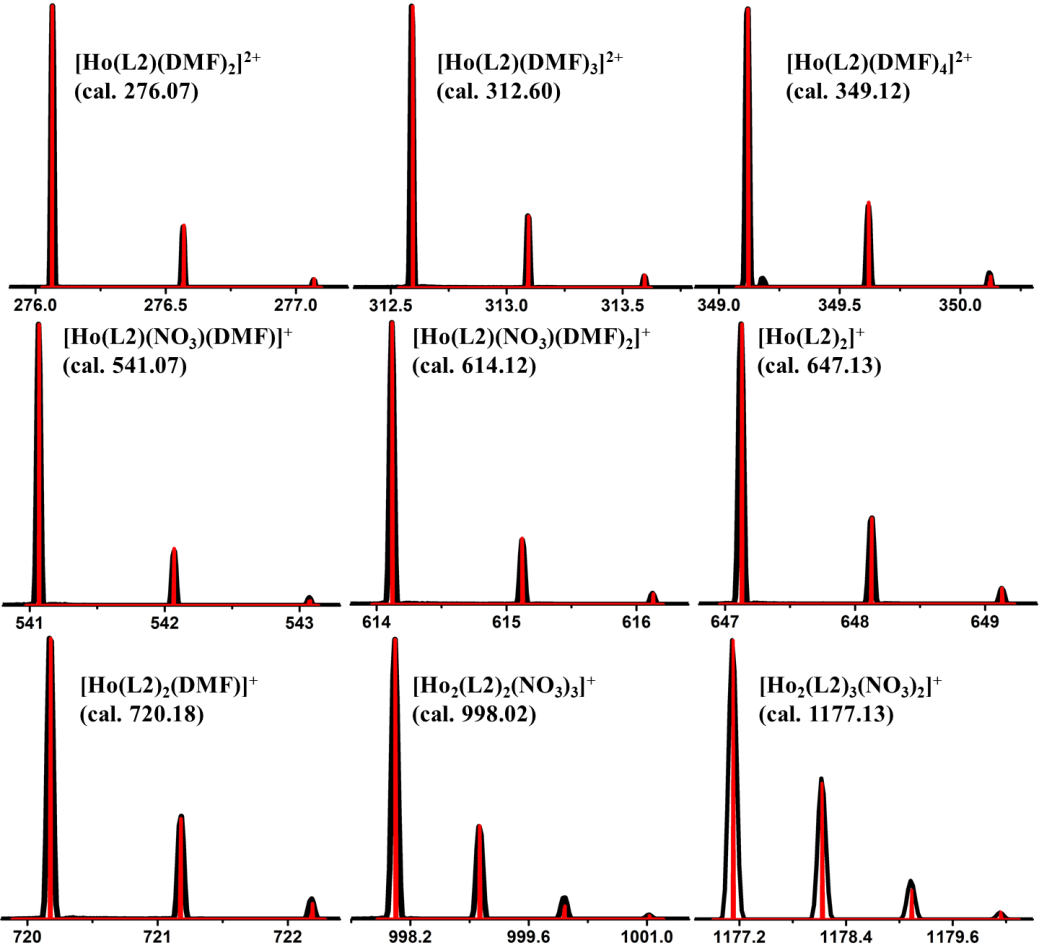


**Figure S7.** The superposed simulated and observed spectra of several species for **3** (In-Source CID 0 eV).


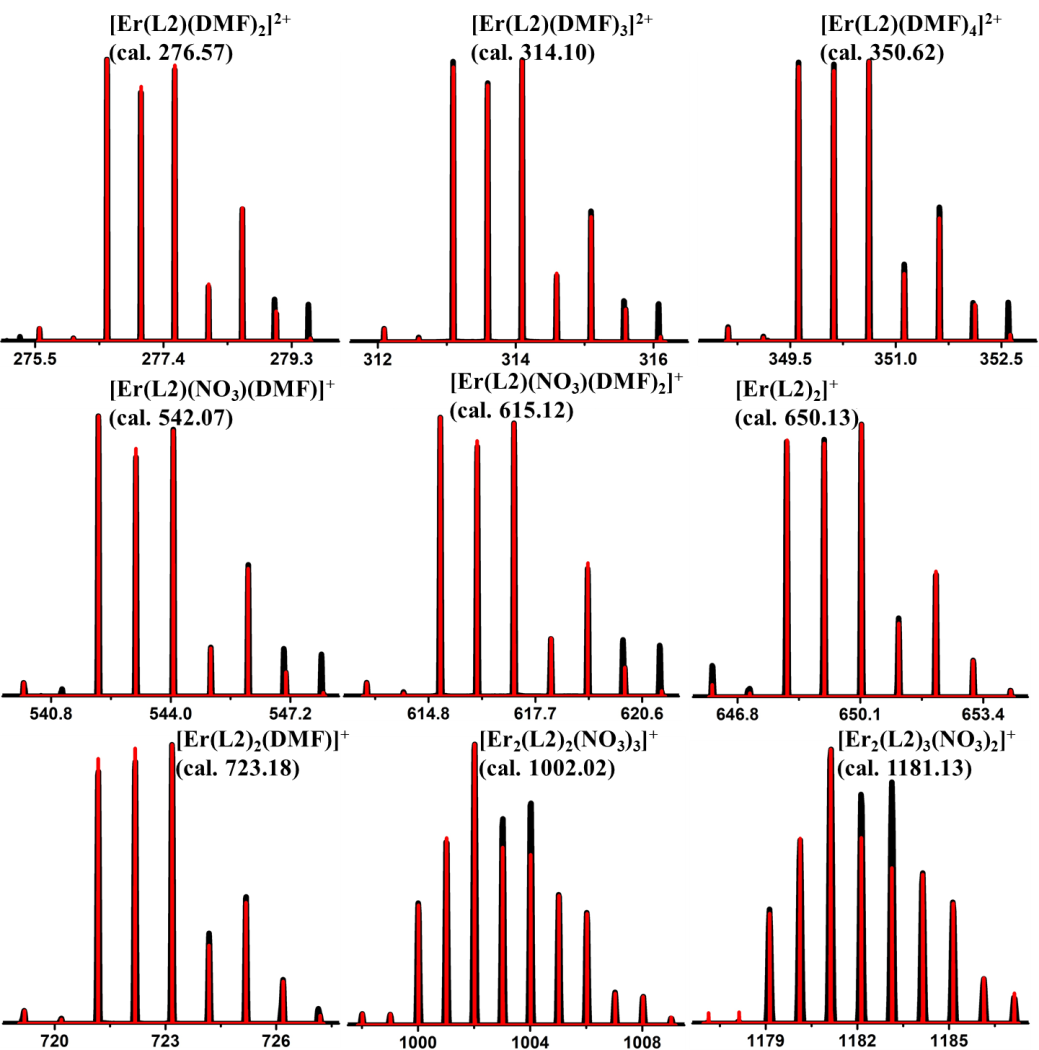


**Figure S8.** The superposed simulated and observed spectra of several species for **4** (In-Source CID 0 eV).


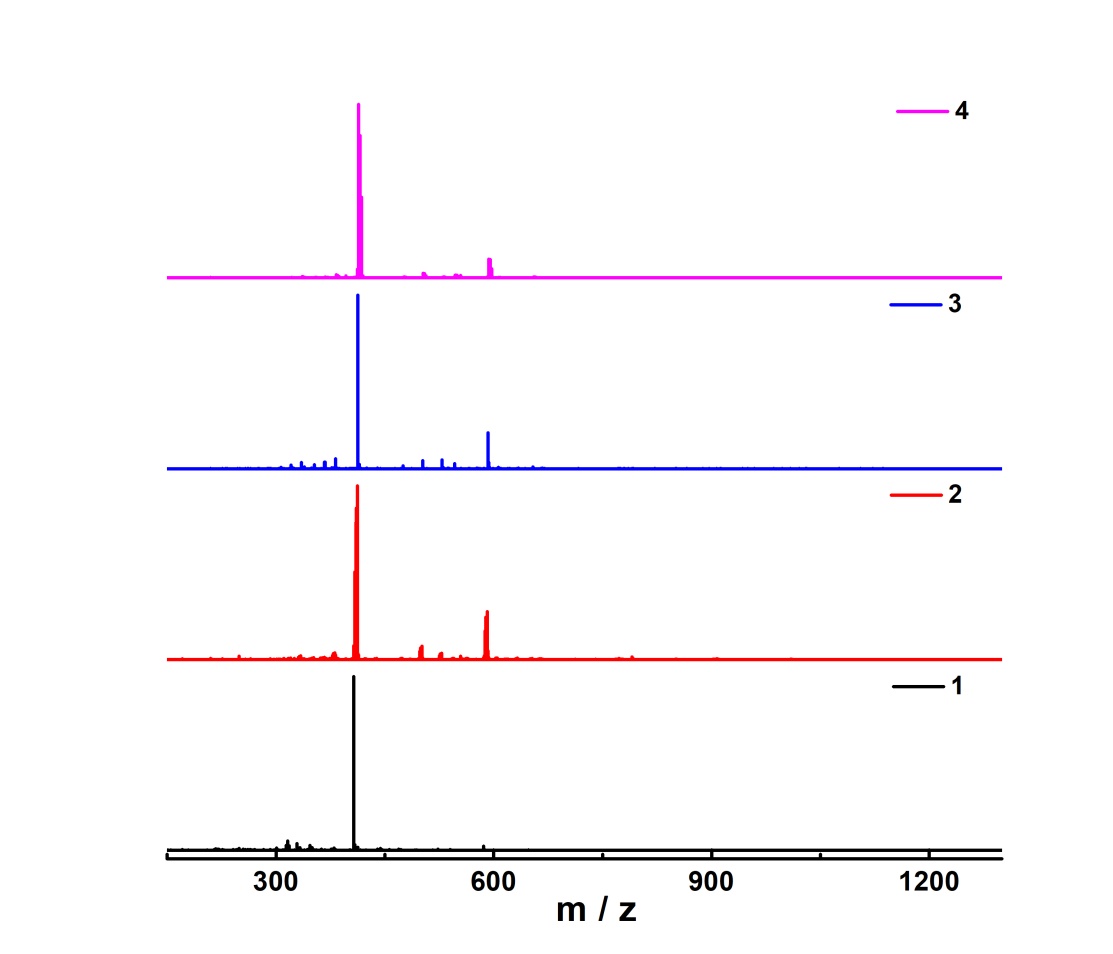


**Figure S9.** Negative ESI-MS spectra of **1**, **2**, **3**, and **4** in CH_3_CN (In-Source CID 0 eV).

**Table S11**. Major species assigned in the ESI-MS of **1**, **2**, **3**, and **4** in negative mode.

| **1** | | | |
| --- | --- | --- | --- |
| Peaks | Relative Intensity | Obs. *m/z* | Calc. *m/z* |
| [Tb(NO_3_)_4_]^-^ | 1 | 406.88 | 406.88 |
| [Tb(L2)(NO_3_)_3_]^-^ | 0.023 | 585.99 | 585.99 |
| **2** | | | |
| [Dy(NO_3_)_4_]^-^ | 1 | 411.88 | 411.88 |
| [Dy(L2)(NO_3_)_3_]^-^ | 590.99 | 0.275 | 590.99 |
| **3** | | | |
| [Ho(NO_3_)_4_]^-^ | 1 | 412.88 | 412.88 |
| [Ho(L2)(NO_3_)_3_]^-^ | 0.206 | 591.99 | 591.99 |
| **4** | | | |
| [Er(NO_3_)_4_]^-^ | 1 | 413.88 | 413.88 |
| [Er(L2)(NO_3_)_3_]^-^ | 0.108 | 592.99 | 592.99 |


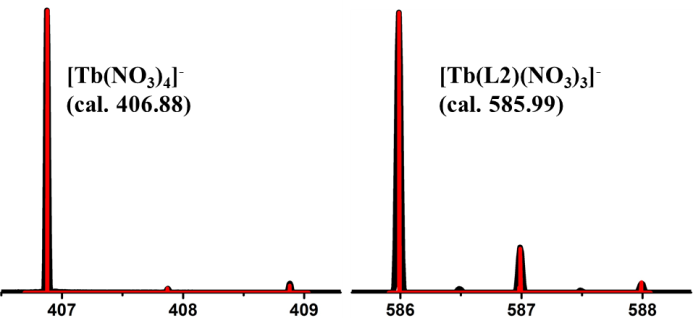


**Figure S10.** The superposed simulated and observed spectra of several species for **1**.


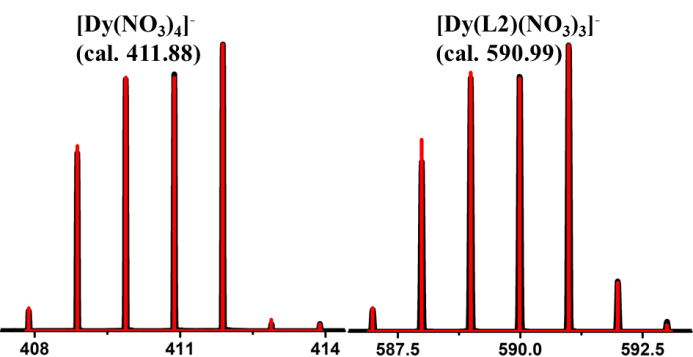


**Figure S11.** The superposed simulated and observed spectra of several species for **2**.


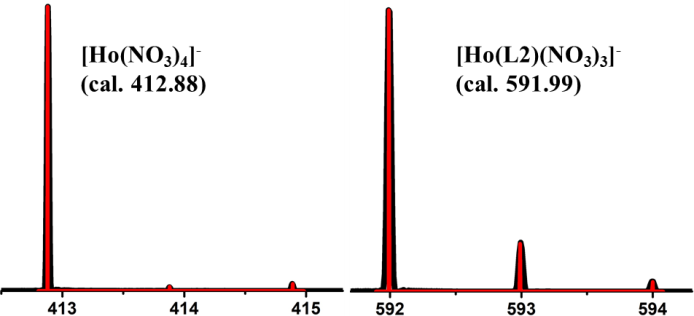


**Figure S12.** The superposed simulated and observed spectra of several species for **3**.


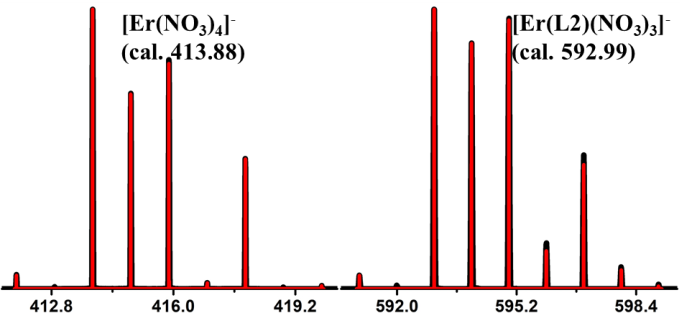


**Figure S13.** The superposed simulated and observed spectra of several species for **4**.


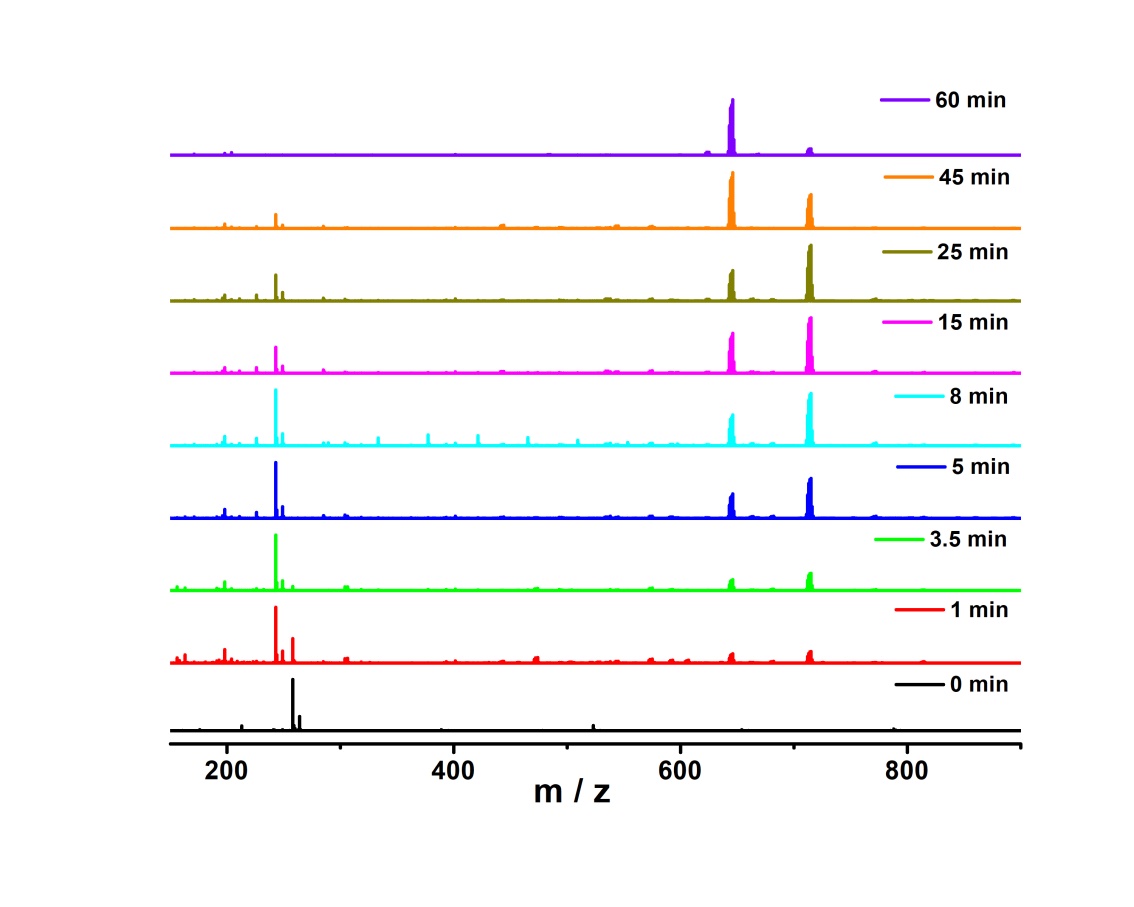


**Figure S14a.** Time-dependent ESI-MS spectra of ligand Li_2_**L1** and 2-aminomethylpyridine reaction with Dy(NO_3_)_3_·6H_2_O in methanol under room temperature conditions.


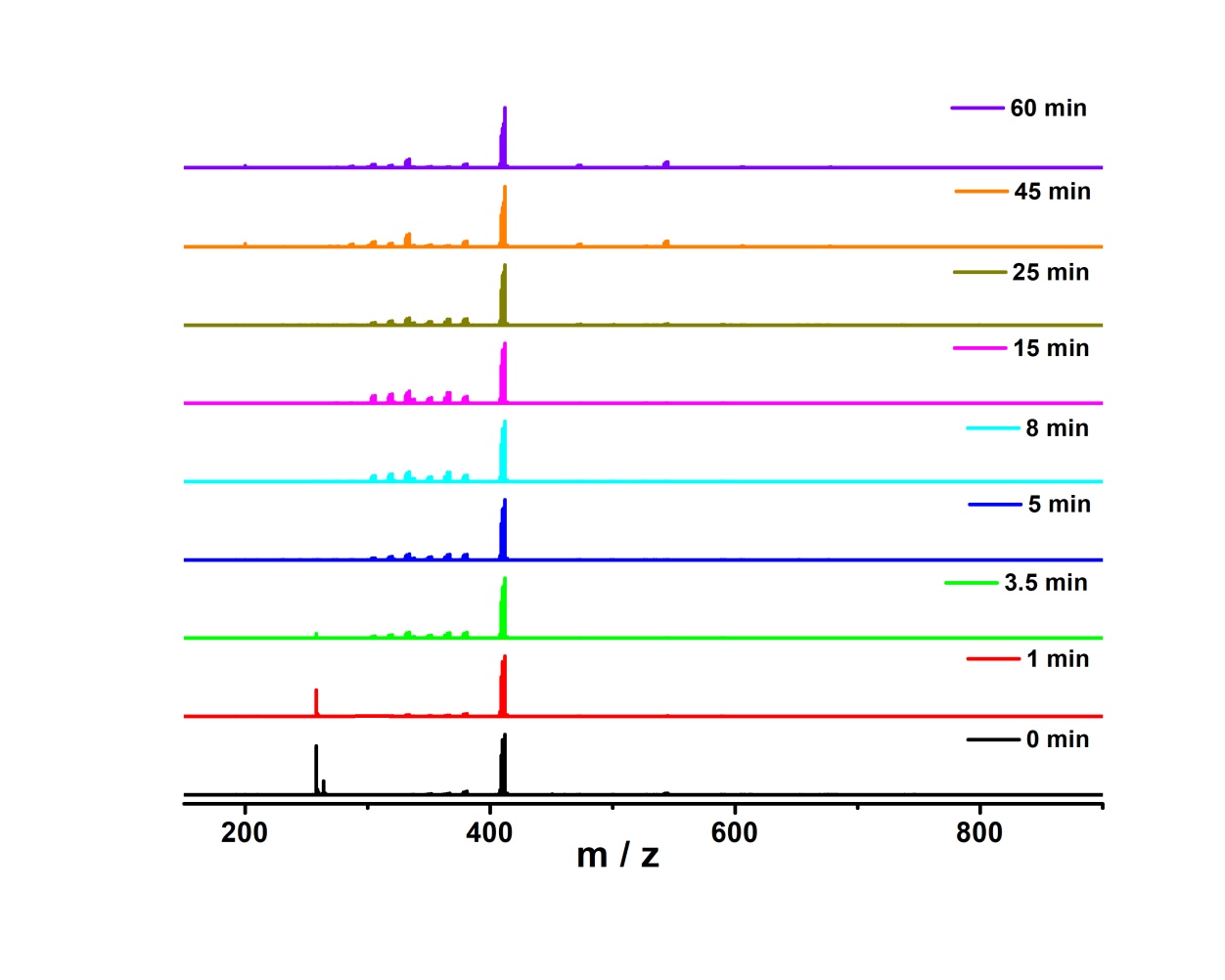


**Figure S14b.** Time-dependent ESI-MS spectra of ligand Li_2_**L1** and 2-aminomethylpyridine reaction with Dy(NO_3_)_3_·6H_2_O in methanol under room temperature conditions in negative mode.

**Table S12**. Time-dependent ESI-MS spectra assigned in the ESI-MS of **2** (Figure S14).

| ***m/z*** | **Fragment** | **Relative Intensity** | | | | | |
| --- | --- | --- | --- | --- | --- | --- | --- |
|  |  | 0min | 1min | 8min | 15min | 45min | 60min |
| 243.11 | [(L2)+2H]^+^ (*calc.* 243.11) | 0 | 1 | 1 | 0.472 | 0.247 | 0 |
| 258.04 | [L1+H]^-^ (*calc.* 258.04) | 1 | 0.437 | 0.077 | 0 | 0 | 0 |
| 646.12 | [Dy(L2)_2_]^+^ (*calc.* 646.12) | 0 | 0.169 | 0.554 | 0.721 | 1 | 1 |
| 715.13 | [Dy(L1)(NO_3_)(solv.)]^+^ (*calc.* 715.12) | 0 | 0.207 | 0.943 | 1 | 0.608 | 0.120 |


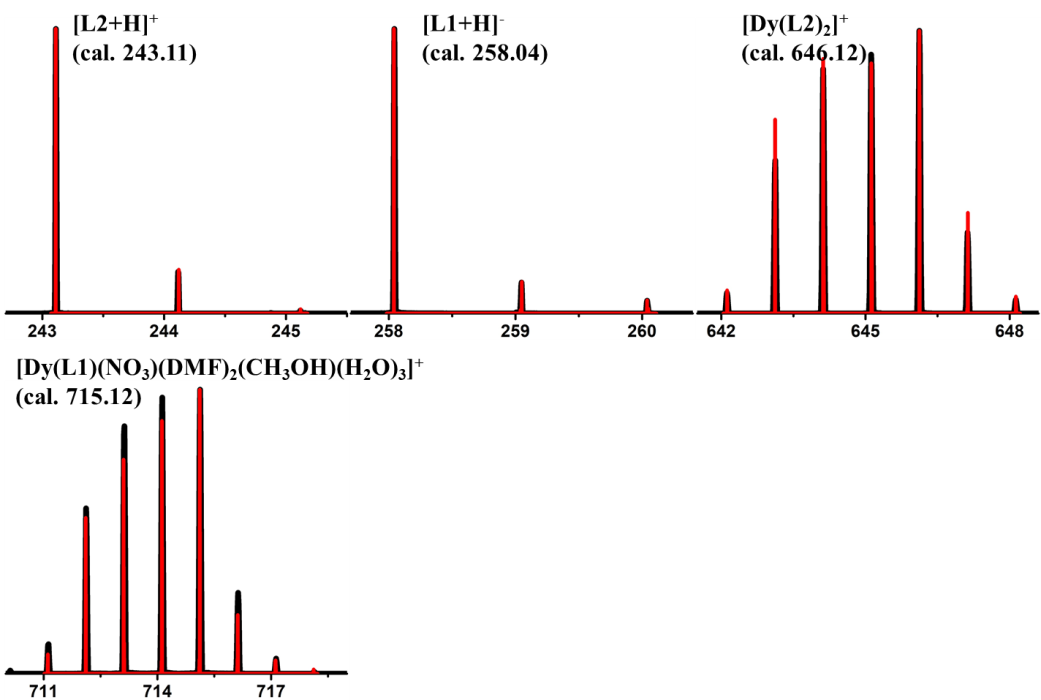


**Figure S15.** The superposed simulated and observed spectra of time-dependent ESI-MS species for **Dy2**.

We observed the luminescence phenomenon with naked eyes under a UV lamp when the reaction process was monitored, therefore, we take a certain amount of **1**, **2**, **3**, and **4** crystals and dissolved them in DMF solution; UV-visible spectroscopy tests were performed separately. **1**, **2**, **3**, and **4** had the same UV absorption peaks (275 and 370 nm) in DMF solution, respectively (Figure S16a). Complexes usually have spectral properties unmatched by general elements due to the special electronic layer structure of the lanthanide metal ions. Therefore, we tested the luminescence of **1**, **2**, **3**, and **4** dissolved in DMF solution at room temperature. When **3** was excited using light with a wavelength of 320 nm, it exhibited a broad emission peak with a wavelength of 445 nm and an emission shoulder with a wavelength of 626 nm (Figure 16b). The broad emission peak at 445 nm is attributed to the π–π* energy-level transition of the organic ligand **L2**. Fluorescence tests were performed on **1** crystals dissolved in DMF solution, which showed broad emission peaks at 444 and 628 nm (Figure 16b). We assign the broad emission peak at 444 nm to the π–π* energy-level transition of the organic ligand **L2**. When we used 315 nm to excite **3** dissolved in DMF, it exhibited a strong emission broad peak wavelength of 445 nm and a weak emission shoulder peak (628 nm) (Figure 16b). The emission peaks at 445 and 628 nm were assigned to the π–π* energy-level transition of the organic ligand **L2**. The complex **4** was excited by a peak with a wavelength of 315 nm to obtain a strong emission broad peak (wavelength of 453 nm) and an extremely weak emission shoulder (wavelength of 625 nm) (Figure 16b). The strong emission peak at 453 nm was attributed to the π–π* energy-level transition of the organic ligand **L2**. The above dinuclear complex was dissolved in DMF and mainly exhibited the luminescent behavior of the organic ligand and the lanthanide metal ion. For **3** and **4**, the luminescent portion of the lanthanide metal ion was masked. To understand the luminescence behavior of the above dinuclear complexes better, we proposed a simple model to explain the luminescence properties (Figure 16c). The energy transfer model shows that Ln(III) ions (Dy(III), Tb(III), and Ho(III)) can accept electrons and energy transfer from the organic ligand **L2** for fluorescence emission. Through the Commission Internationale de L'Eclairage (CIE) color coordinates, we can see that **Ln2** (Ln = Tb, Dy, Ho, Er) mainly emits a blue-green light in DMF solution (Figure 16c). The color coordinates of the above Ln(III) complex in the CIE color diagram are **1** (0.220, 0.164), **2** (0.226, 0.182), **3** (0.163, 0.215), and **4** (0.176, 0.132), respectively. To further investigate the **Ln2** luminescence behavior, we tested its solid state luminescence (Figure S17). In the solid state, **2** shows a strong emission peak at 484 nm and 575 nm, and it emits yellow-green light. **1** showed a strong spike emission at 425 nm. **3** and **4** show strong peak emission at 529 nm and 560 nm, respectively. To further determine the luminescence assignment of **Ln2**, here we tested the photoluminescence of ligand **L2** dissolved in DMF at an excitation wavelength of 317 nm, which shows a strong emission broad peak with a maximum emission wavelength of 485 nm and photoluminescence behavior was not shown at 626 nm (Figure S17).


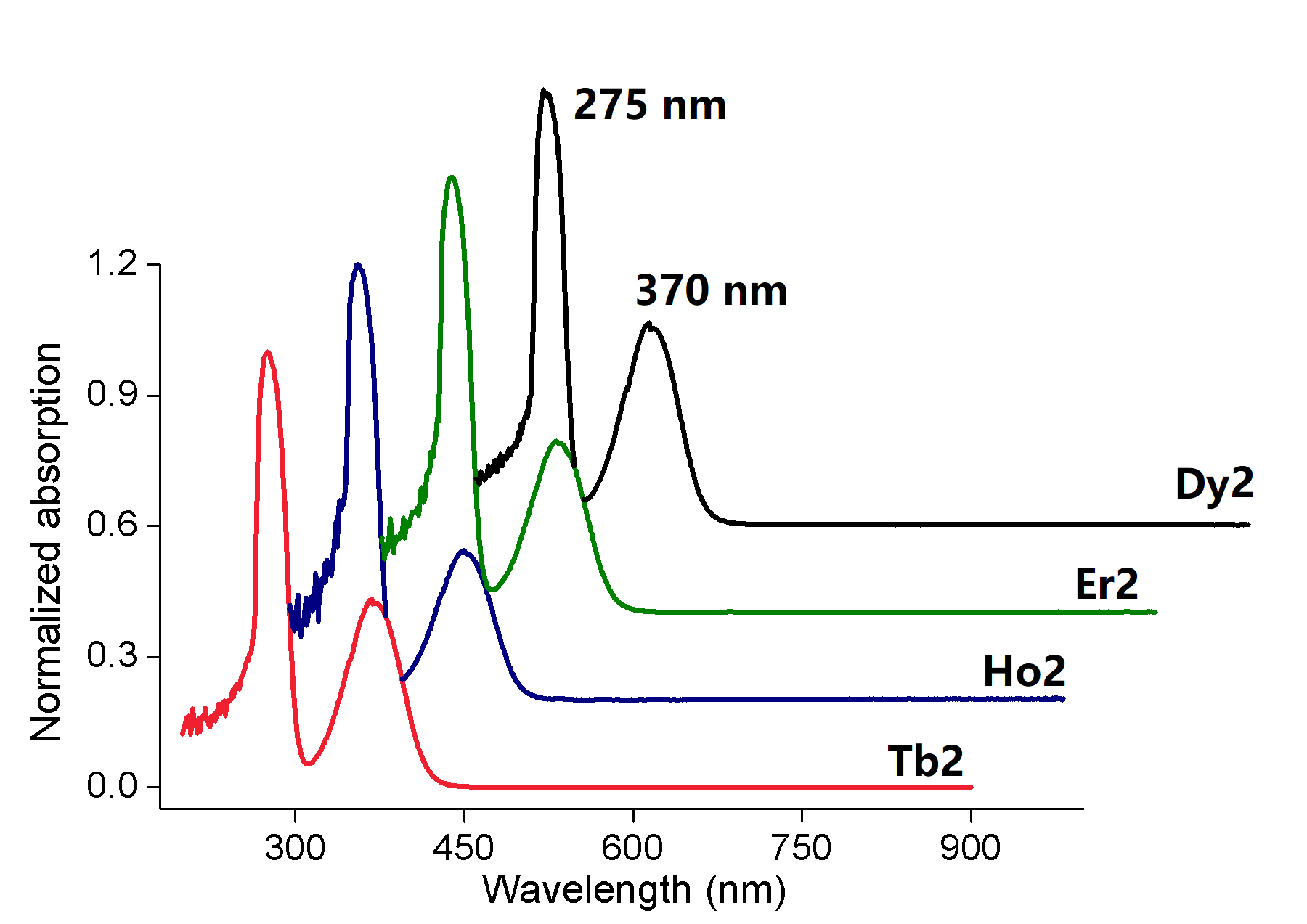


**Figure S16a.** The **Ln2** (**1**, **Tb2**; **2**, **Dy2**; **3**, **Ho2**; and **4**, **Er2**) complex was dissolved in an ultraviolet-visible absorption test in DMF, respectively.


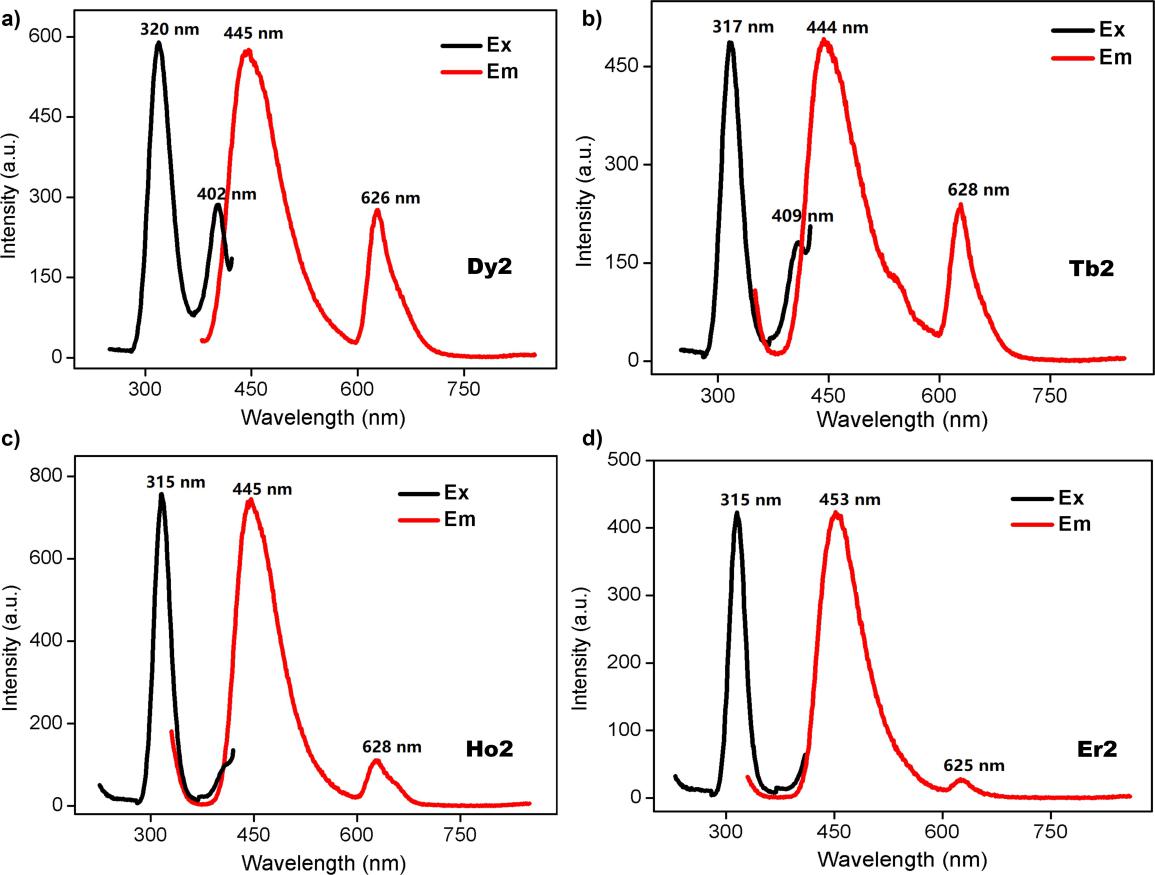


**Figure 16b**. The dinuclear compounds **2** (a), **1** (b), **3** (c), **4** (d) were dissolved in DMF solution for luminescence (excitation and emission) tests, respectively.


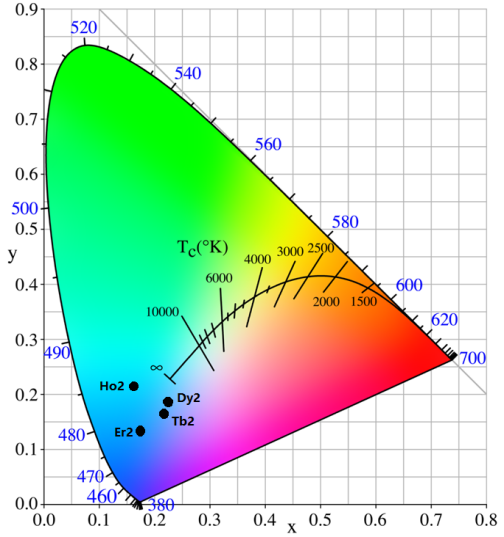


**Figure 16c**. CIE chromaticity map.


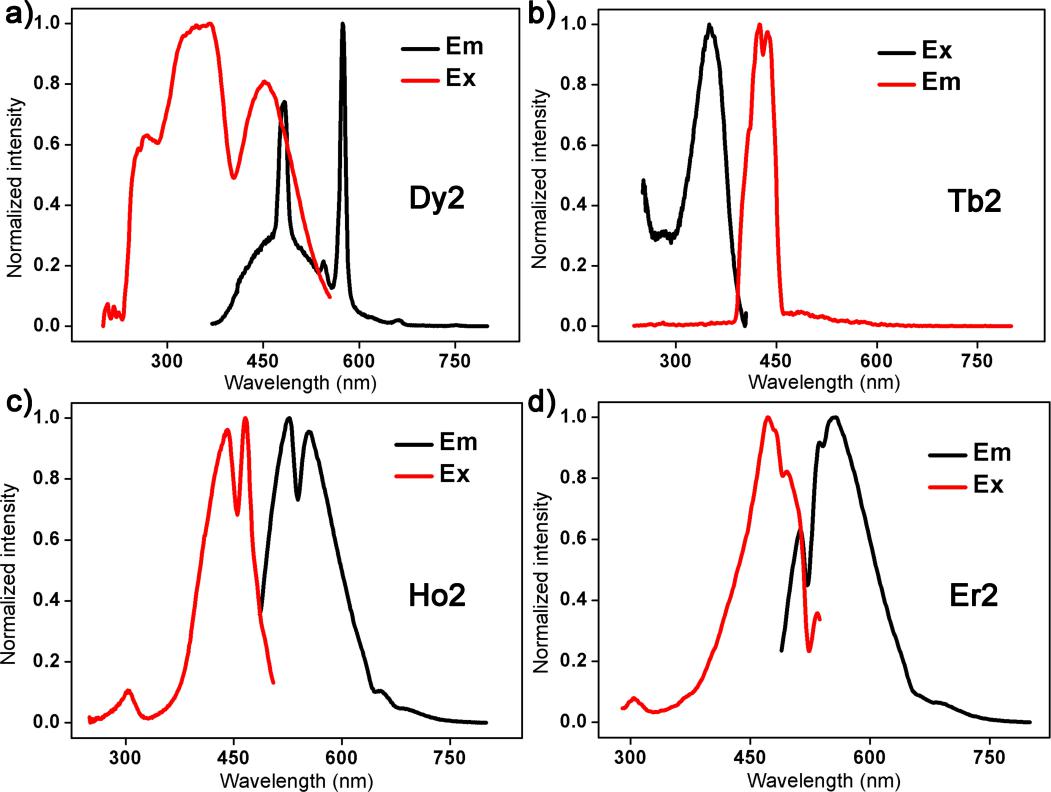


**Figure S17a.** Photoluminescence in **Ln2** solid state conditions.


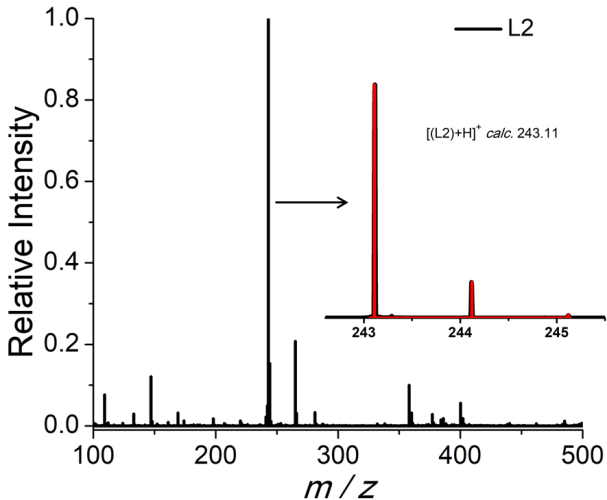


**Figure S17b.** The **L2** was dissolved in DMF solution for ESI-MS measurement.


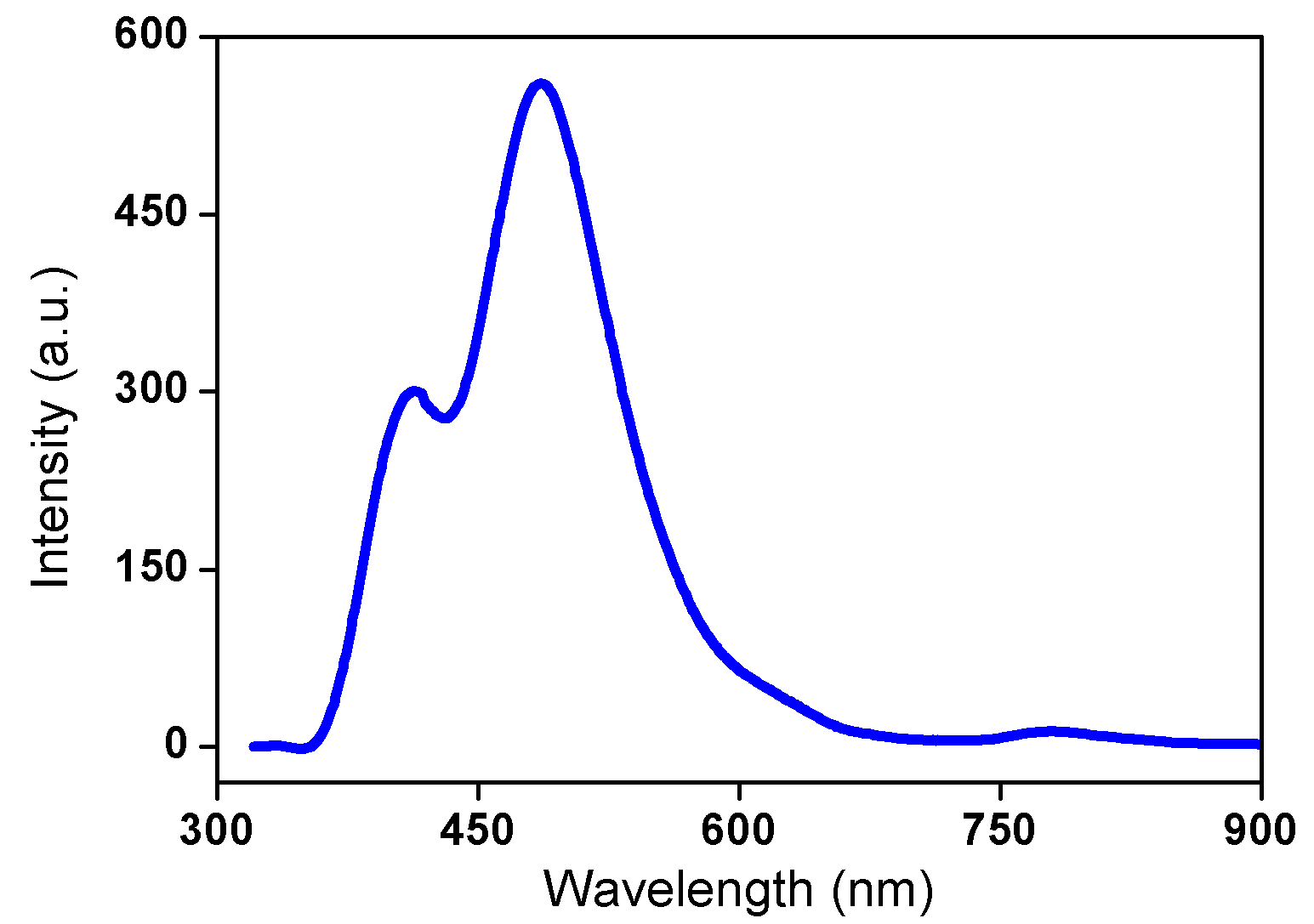


**Figure S17c.** The **L2** was dissolved in DMF solution for luminescence tests (emission).


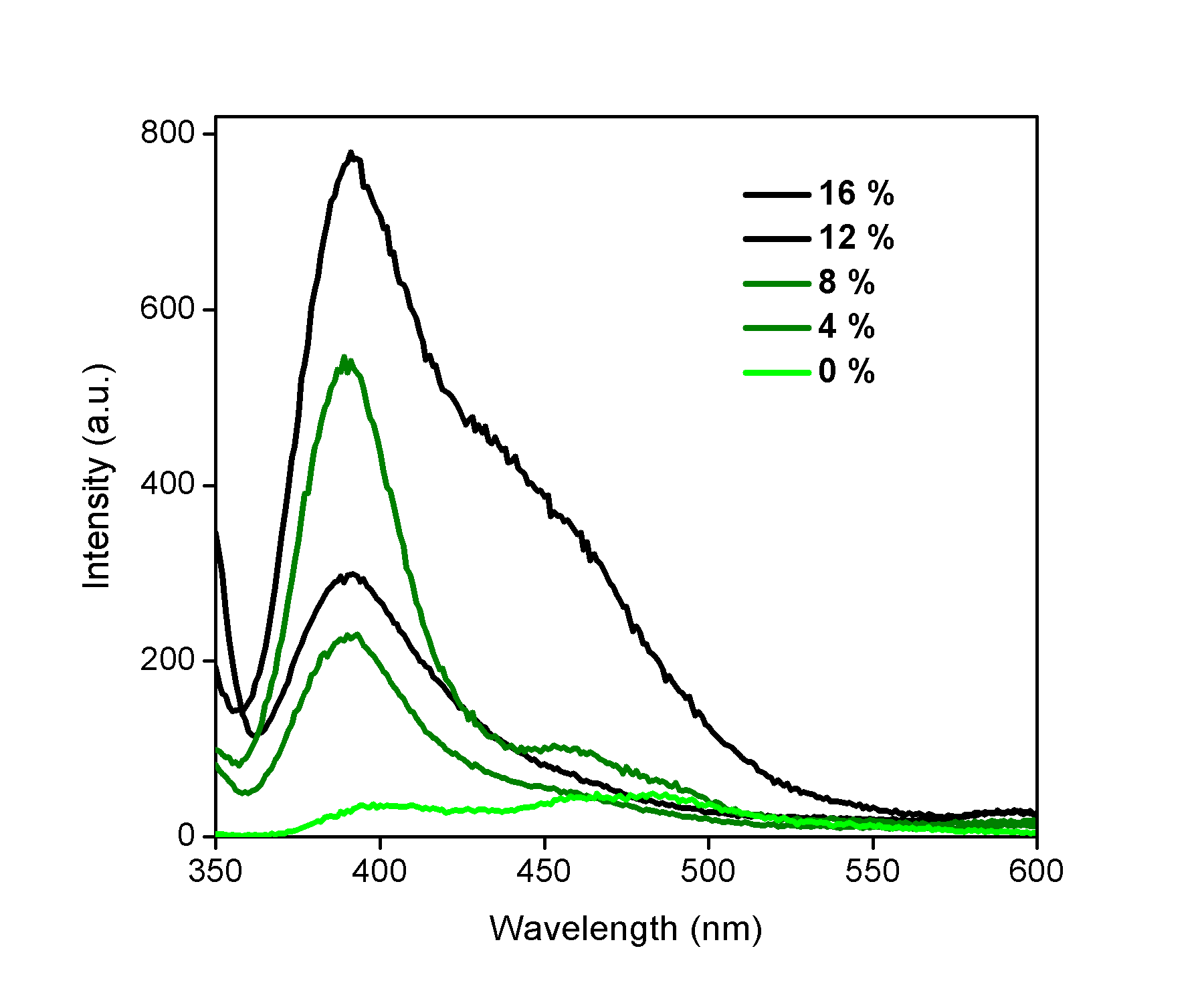


**Figure S18.** The luminescence spectra of **1** dispersed in H_2_O with increasing CH_2_Cl_2_ content (CH_2_Cl_2_/ H_2_O).


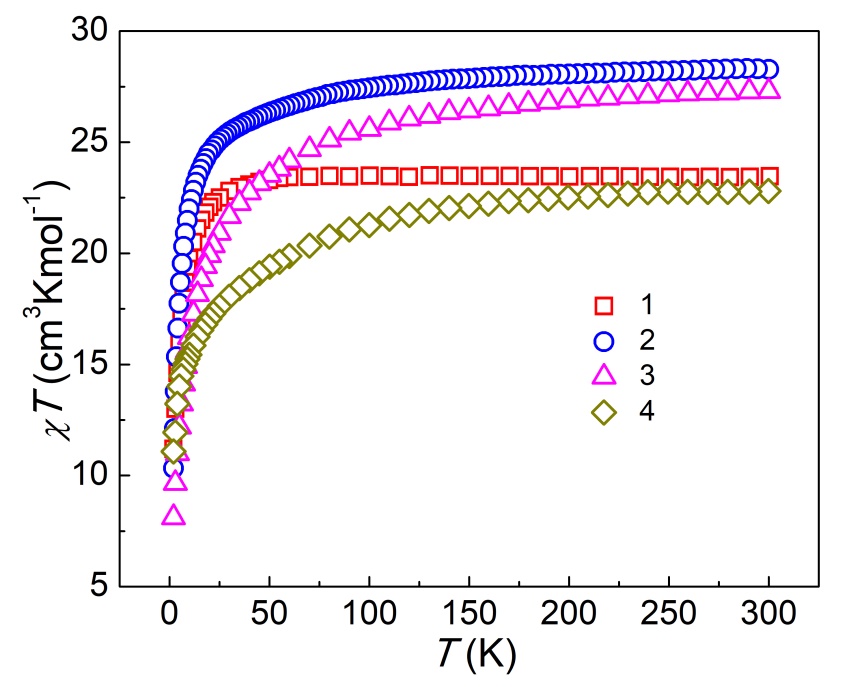


**Figure S19.** Temperature dependence of *χ_m_T* for all complexes.


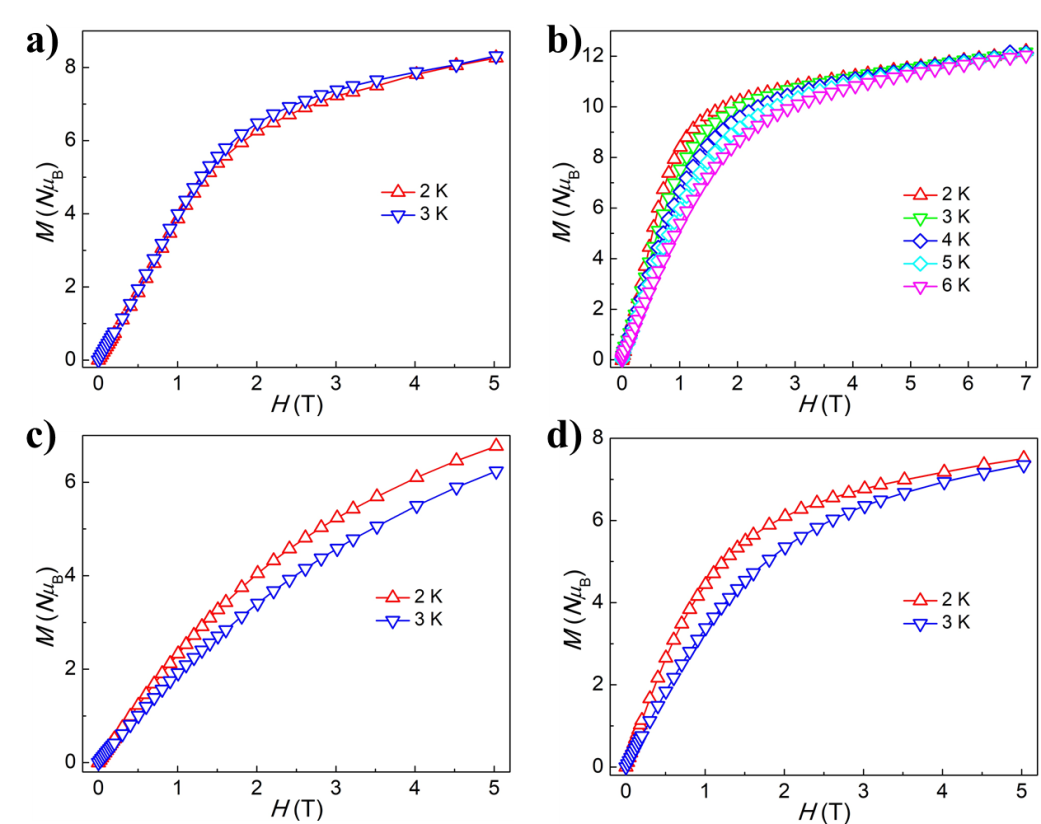


**Figure S20**. *M* vs. *H* plots for **1** (a), **2** (b), **3** (c) and **4** (d).


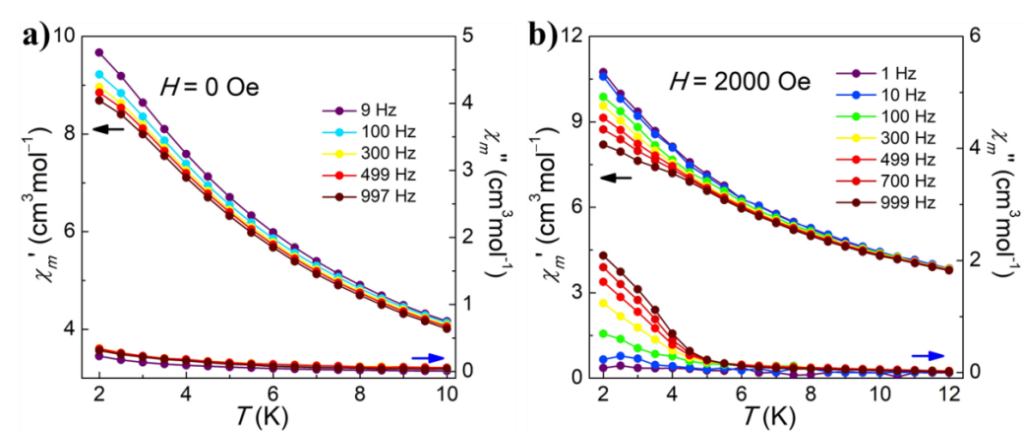


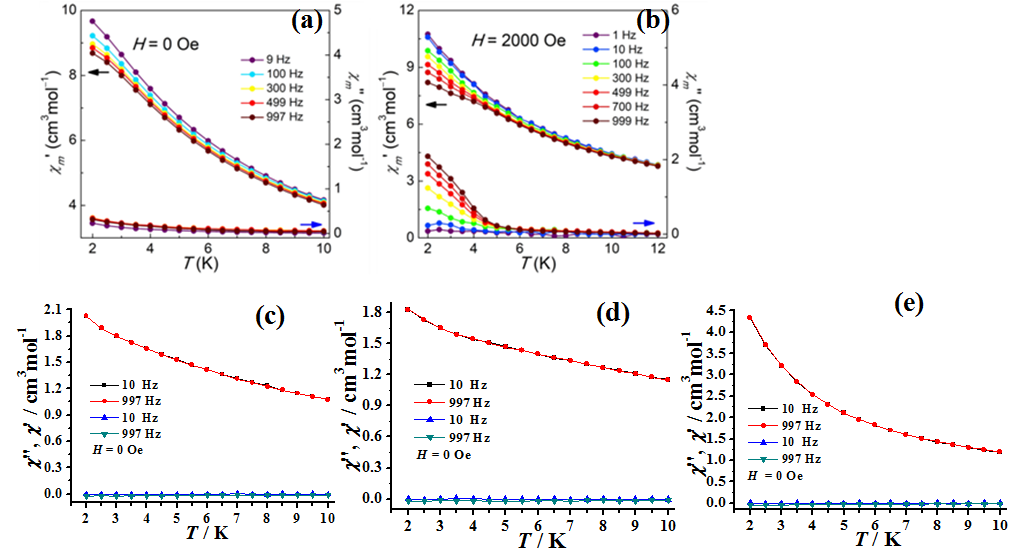


**Figure S21**. The temperature dependence of the in-phase and out-of-phase ac-susceptibilities for different frequencies for **2** (a, b), **1** (c), **3** (d) and **4** (e).


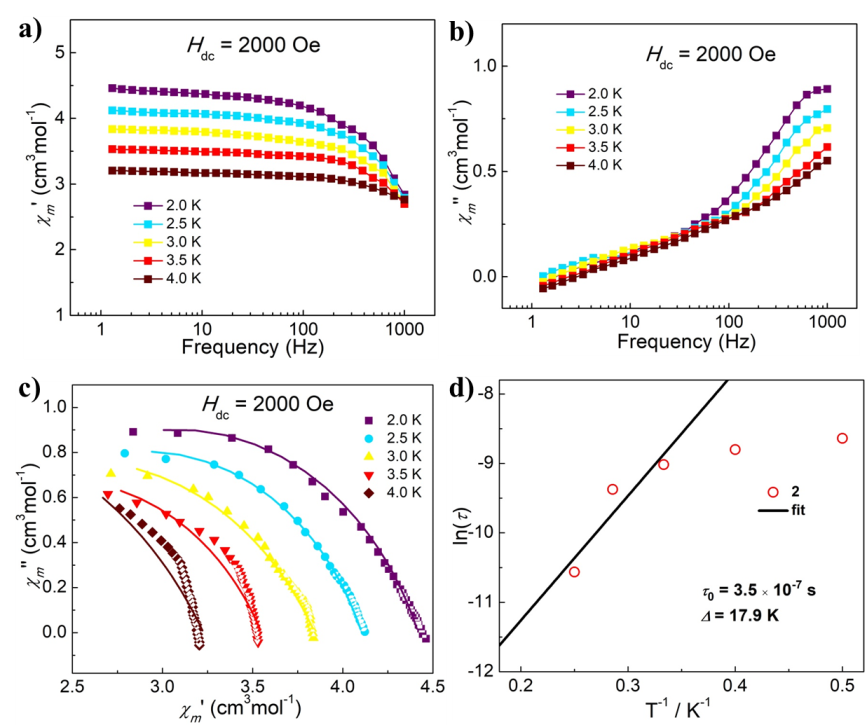


**Figure S22**. Variable–frequency dependence of AC susceptibilities (a and b) under 2000 Oe dc field; Cole–Cole plots of AC susceptibilities for **2** (c); (d) and Arrhenius plots generated from the temperature-dependent relaxation times extracted from the ac-susceptibilities Cole–Cole fits.

**Table S13.** Selected parameters from the fitting results of the Cole-Cole plots for **2** under 2000 Oe field.

|  | **2** | | |
| --- | --- | --- | --- |
| *Temp.*(K) | *τ* | *α* | residual |
| 2.0 | 1.77E-04 | 2.43E-01 | 8.10E-02 |
| 2.5 | 1.51E-04 | 2.48E-01 | 8.51E-02 |
| 3.0 | 9.28E-05 | 3.25E-01 | 9.58E-02 |
| 3.5 | 8.48E-05 | 3.02E-01 | 1.17E-01 |
| 4.0 | 2.59E-05 | 3.36E-01 | 1.71E-01 |


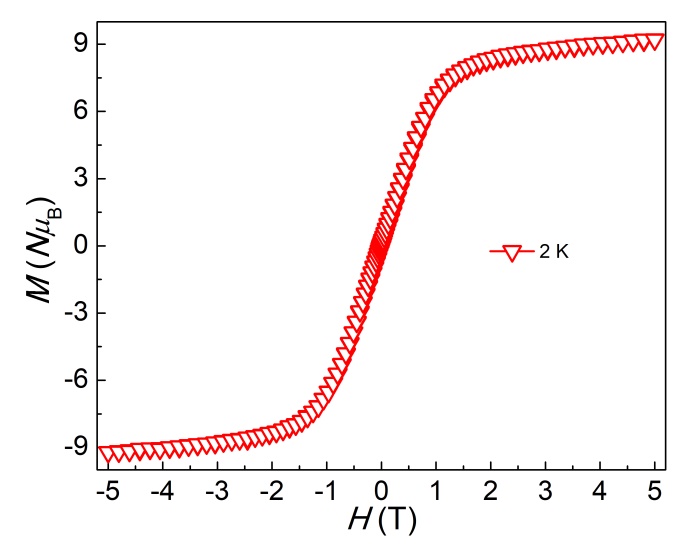


**Figure S23**. Hysteresis loop for **2** at 2 K.
